# The benefits and risks of menopause hormone therapy for the cardiovascular system in postmenopausal women: a systematic review and meta-analysis

**DOI:** 10.1186/s12905-023-02788-0

**Published:** 2024-01-23

**Authors:** Yimeng Gu, Fangfang Han, Mei Xue, Miyuan Wang, Yuxiao Huang

**Affiliations:** 1 Xiyuan Hospital, China Academy of Chinese Medical Sciences, Beijing, 100091 China; 2 National Clinical Research Center for Chinese Medicine Cardiology, Beijing, 100091 China; 3 School of Public Health, Tongji Medical College, Huazhong University of Science and Technology, Wuhan, 430030 China

**Keywords:** Menopause, Cardiovascular, Menopause hormone therapy, Thrombosis, Stroke, Arterial dilation, Meta-analysis

## Abstract

**Background:**

Menopause hormone therapy (MHT), as an effective method to alleviate the menopause-related symptoms of women, its benefits, risks, and potential influencing factors for the cardiovascular system of postmenopausal women are not very clear.

**Objectives:**

To evaluate cardiovascular benefits and risks of MHT in postmenopausal women, and analyze the underlying factors that affect both.

**Search strategy:**

The EMBASE, MEDLINE, and CENTRAL databases were searched from 1975 to July 2022.

**Selection criteria:**

Randomized Clinical Trials (RCTs) that met pre-specified inclusion criteria were included.

**Data collection and analysis:**

Two reviewers extracted data independently. A meta-analysis of random effects was used to analyze data.

**Main results:**

This systematic review identified 33 RCTs using MHT involving 44,639 postmenopausal women with a mean age of 60.3 (range 48 to 72 years). There was no significant difference between MHT and placebo (or no treatment) in all-cause death (RR = 0.96, 95%CI 0.85 to 1.09, I^2^ = 14%) and cardiovascular events (RR = 0.97, 95%CI 0.82 to 1.14, I^2^ = 38%) in the overall population of postmenopausal women. However, MHT would increase the risk of stroke (RR = 1.23, 95%CI 1.08 to 1.41,I^2^ = 0%) and venous thromboembolism (RR = 1.86, 95%CI 1.39 to 2.50, I^2^ = 24%). Compared with placebo, MHT could improve flow-mediated arterial dilation (FMD) (SMD = 1.46, 95%CI 0.86 to 2.07, I^2^ = 90%), but it did not improve nitroglycerin-mediated arterial dilation (NMD) (SMD = 0.27, 95%CI − 0.08 to 0.62, I^2^ = 76%). Compared with women started MHT more than 10 years after menopause, women started MHT within 10 years after menopause had lower frequency of all-cause death (*P* = 0.02) and cardiovascular events (*P* = 0.002), and more significant improvement in FMD (*P* = 0.0003). Compared to mono-estrogen therapy, the combination therapy of estrogen and progesterone would not alter the outcomes of endpoint event. (all-cause death *P* = 0.52, cardiovascular events *P* = 0.90, stroke *P* = 0.85, venous thromboembolism *P* = 0.33, FMD *P* = 0.46, NMD *P* = 0.27).

**Conclusions:**

MHT improves flow-mediated arterial dilation (FMD) but fails to lower the risk of all-cause death and cardiovascular events, and increases the risk of stroke and venous thrombosis in postmenopausal women. Early acceptance of MHT not only reduces the risk of all-cause death and cardiovascular events but also further improves FMD, although the risk of stroke and venous thrombosis is not reduced. There is no difference in the outcome of cardiovascular system endpoints between mono-estrogen therapy and combination therapy of estrogen and progesterone.

**Supplementary Information:**

The online version contains supplementary material available at 10.1186/s12905-023-02788-0.

## Introduction

Over the past 20 years, cardiovascular disease has ranked first among the top 10 causes of death in the world [1]. The risk of cardiovascular disease varies by age in men and women, with the average age of onset time in women being about 10 years behind that of men, although the overall risk in both them is roughly the same [2]. Compared with premenopausal peers, the incidence of cardiovascular events in postmenopausal women increases 1.6 times [3]. Most healthy women enter perimenopause around the age of 50, and about 75% of them will have menopause-related symptoms, such as vasomotor syndrome (VMS), genitourinary syndrome of menopause (GSM), etc. [4] Menopause hormone therapy (MHT), as the most common and effective treatment to relieve postmenopausal symptoms [5], has been in the spotlight since the 70s of last century. Observational studies showed that long-term MHT was beneficial because it could reduce the risk of cardiovascular disease, osteoporosis, etc. [6, 7]. However, results from randomized controlled trials (RCTs) represented by the Women’s Health Initiative which was published in 2002, showed that MHT did not reduce the risk of all-cause death and cardiovascular events (cardiovascular death and non-fatal myocardial infarction) in postmenopausal women, the incidence of stroke and venous thromboembolism increased significantly, and the overall risk outweighed the benefits [8]. These contradictory conclusions led to the emergence of “time hypothesis”: Inconsistence in risk of cardiovascular disease between diverse clinical studies seemed can be explained by different onset time of MHT [9, 10]. The positive results from observational studies might be due to the fact that subjects started MHT shortly after menopause, while the subjects included in RCTs began to receive MHT at 5 to 20 years after menopause. The Danish Osteoporosis Prevention Study (DOPS) published on British Medical Journal (BMJ) in 2012 showed that starting MHT in the early stage of menopause could reduce the incidence of composite endpoints of heart failure, myocardial infarction, and all-cause death in postmenopausal women, which has aroused widespread attention in academia [11]. The study was followed up for 16 years, and the mean baseline age of subjects (49.7 ± 2.8 years) was younger than other similar clinical studies.

To further verify the explanatory role of the “time hypothesis” in different studies and explore other underlying factors that might lead to contradictory conclusions, and with the aim of better understanding the benefits and risks of MHT on the cardiovascular system of postmenopausal women, we conducted this systematic review and meta-analysis of six variables related to cardiovascular risk reported by RCTs: all-cause death, cardiovascular events (cardiovascular death and non-fatal myocardial infarction), stroke, venous thromboembolism, flow-mediated arterial dilation (FMD), and nitroglycerin-mediated arterial dilation (NMD). These data were obtained from postmenopausal women undergoing MHT at different ages.

## Methods

This review was designed according to the guidelines of PRISMA (Preferred Reporting Items for Systematic Reviews and Meta-Analysis) and MOOSE (Meta-analysis of Observational Studies in Epidemiology). The approaches of Cochrane and GRADE (Grading of Recommendations, Assessment, Development, and Evaluation) were used to guide the conduct of this systematic review and the credibility of evidence for outcomes. This review was prospectively registered in the PROSPERO database (registration number: CRD42022368553) on October 30, 2022.

### Eligibility criteria, information sources, search strategy

EMBASE, MEDLINE, and Cochrane Central Register of Controlled Trials (CENTRAL), the above three databases, without language restriction, were used to search for RCTs from 1975 to July 2022 performed in postmenopausal women receiving MHT (mono-estrogen therapy or combination therapy of estrogen and progesterone). Specific search strategies were described in Methods S1. Original studies met the following criteria were included: (1) RCTs; (2) included a control group (placebo or no treatment); (3) studied the effects of MHT on all-cause death, cardiovascular events, stroke, venous thromboembolism, FMD and NMD; (4) studies on all-cause death, cardiovascular events, stroke, and venous thromboembolism, the follow-up time must be ≥1 year, and the number of subjects must not be less than 80, but no such limitation in studies related to FMD and NMD. Exclusion criteria: (1) observational research; (2) the subjects were premenopausal women; (3) MHT studies related to phytohormone therapy.

### Study selection

Two reviewers (Gu YM and Han FF) independently screened the titles and abstracts of the relevant literature in the database search results. The full text of any literature considered to be eligible for systematic review was obtained, and the relevance of each paper to this review was independently evaluated according to the pre-established review criteria. Disagreements between two reviewers were resolved by consulting relevant literature or communicating with Huang YX.

### Data extraction

Outcomes of interest included all-cause death, cardiovascular events (cardiovascular death and non-fatal myocardial infarction), stroke, venous thromboembolism, FMD, and NMD. To intuitively show the effects of MHT on arterial dilation, FMD and NMD were included in the analysis as the percentage changes in brachial artery diameter: [(post-interventional brachial artery diameter – pre-interventional brachial artery diameter) / pre-interventional brachial artery diameter] × 100%. We used the Review Manager (RevMan5.4.1) to conduct the meta-analysis. Peto modified Mantel-Haenszel method and random-effects model were used to provide an overall estimate of the therapeutic effect. The dichotomous variables were reported as risk ratio (RR) and 95% confidence intervals (CIs). Continuous variables were reported as standardized mean differences (SMDs) and 95%CIs. For time-event outcome data of stroke, we extracted patient-level time-event data by digitizing the Kaplan Meier curve [12] and confirmed consistency with the values in the original study report. Meanwhile, the Cox regression model was used to fit the relationship between survival distributions and different interventions, and hazard ratio (HR) and 95%CIs were reported. The bilateral *P* value < 0.05 was considered statistically significant. GetData Graph Digitizer 2.24 (http://getdatagraph-digitizer.com) was applied to digitalize and extract the data when the included study did not provide specific data but only presented it as graphs. Two reviewers (Gu YM and Han FF) extracted data independently and resolved the divergences by consulting relevant literature or communicating with Huang YX. In our analysis, multiple reports of the same trial (e.g. different follow-up time points or subgroup analysis) were considered as a single trial. Conversely, studies presenting two different trials or comparisons in one design were considered as two separate individual trials. Finally, relevant data was pooled together and supplemented with sensitivity analysis. Besides, the constant continuous correction method was used for the zero-event trials.

### Assessment of bias risk

We evaluated the risk of bias according to the assessment criteria in the *Cochrane Handbook for Systematic Reviews of Interventions* [13] (quality of random sequence generation and allocation, blinding, incomplete result data, selective result reports, and other sources of bias). The assessments of bias risk were conducted independently by two reviewers (Gu YM and Han FF). Lastly, the GRADE approach [14] (risk of bias, imprecision, inconsistency, indirectness, publication bias, and others) was used to evaluate the reliability of each outcome evidence, and trial sequential analysis (TSA-0.9.5.10-Beta) was applied as an objective measure of the required information size (RIS) and accuracy [15].

### Data synthesis

This study used the Chi2 test and I^2^ statistic to quantitatively explore the heterogeneity. The *P* value obtained by Chi2 test < 0.10 indicated significant statistical heterogeneity between the trials. 0 to 40% of I^2^ statistic was considered as potentially unimportant; 30 to 60% represented moderate heterogeneity; 50 to 80% represented substantial heterogeneity; 70 to 100% represented considerable heterogeneity. Publication bias was assessed by examining the funnel plots. To evaluate the impact of each study on the overall effect size, sensitivity analysis was carried out using the leave-one-out method (one study was removed and the analysis was repeated each time) [16–18], and the potential factors affecting the overall therapeutic effect were further explored through subgroup analysis.

### Subgroup analysis

To assess the potential effects of “time hypothesis” on the cardiovascular system in postmenopausal women, we stratified trials according to the onset time of MHT (< 10 years or ≥ 10 years after menopause). If these data were not available, the mean age of subjects at baseline (≤ 60 years or ≥ 65 years) was used as a substitute. In addition, to explore the impact of subjects’ health status and different treatment protocols on the endpoint events included in the study, we grouped the data according to whether the treatment measures were primary or secondary prevention and protocols of treatment as mono-estrogen or combination of estrogen and progesterone.

## Results

**Fig. 1:**
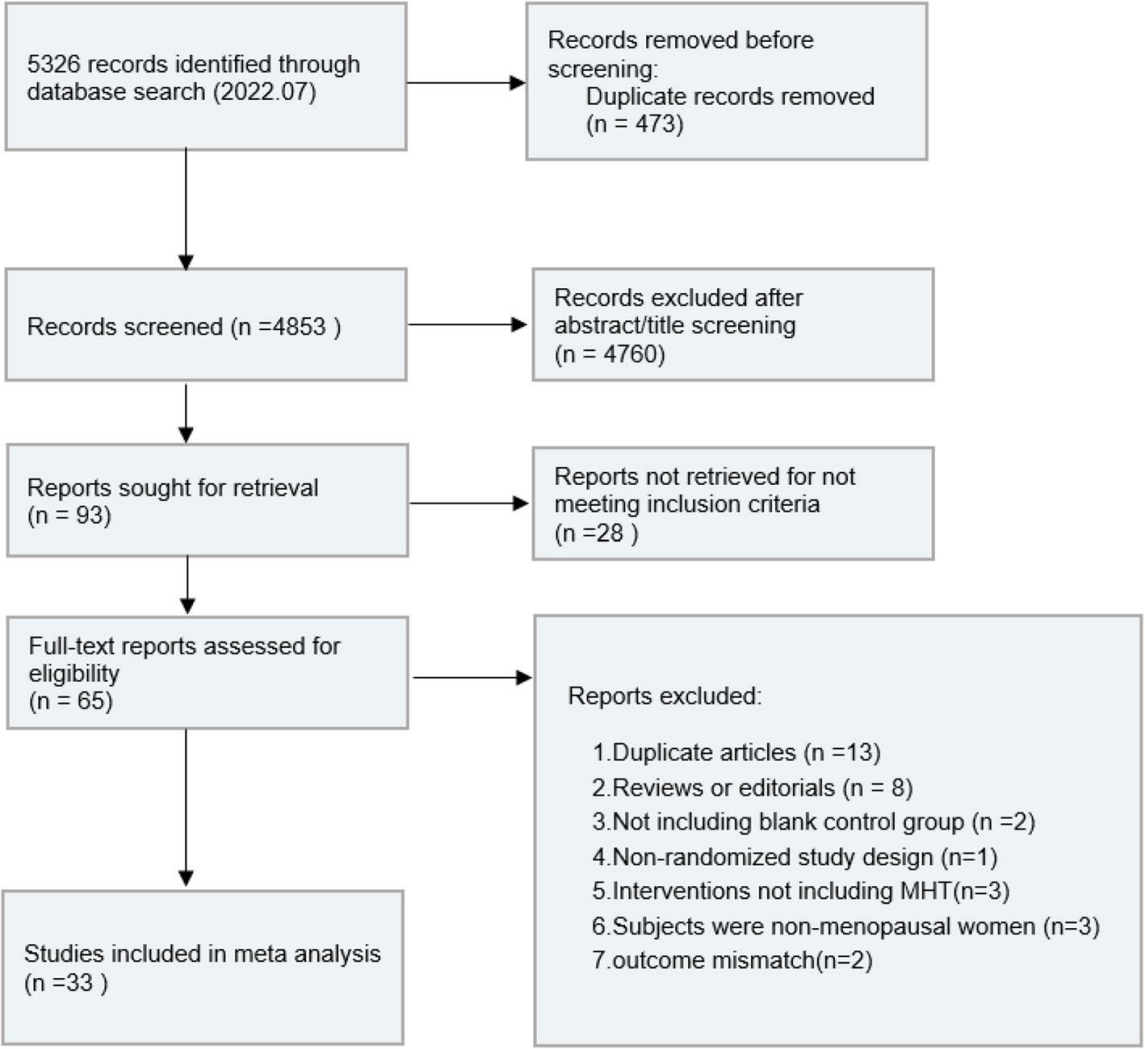
Study selection process for systematic review and meta-analysis

4853 records were identified in this systematic review. Among the 65 full-text articles assessed for eligibility, 32 studies were excluded because of the following reasons: duplicated articles (*n* = 13), reviews or editorials (*n* = 8), no blank control group (*n* = 2), non-randomized trial design (*n* = 1), interventions not including MHT (*n* = 3), and subjects were non-menopausal women (*n* = 3), outcome mismatch (*n* = 2). 33 RCTs with a total of 44,639 subjects that finally met the evaluation criteria were included (Fig. 1) [8, 11, 19–49].

### Description of included studies

The included studies were published from July 1979 to May 2020. All these studies used estrogen drugs. The subjects were all postmenopausal women, with an average age of 60.3 (range 48 to 72 years). In addition, the single therapeutic doses ranged from 10μg to 4 mg, and the duration of treatment varied from 2 hours to 16 years. The interventions included in studies and the baseline characteristics of subjects are shown in Table 1.

**Table 1:** Characteristics of studies and population baseline

Study	Publish date,year	No.of patients	Group:average age(SD/range), Years	Intervention groups	Control	Follow up time	Evaluated outcomes
David M et al	2000	309	E:66.3(7.6) E + P:65.6(6.5) C:65.6(7.3)	0.625 mg conjugated estrogen+ 2.5 mg medroxyprogesterone acetate	Placebo	3.2 y	All-cause death, CVE,stroke,VT
DOPS	2012	1006	E/E + P:50(2.8) C:49.5(2.7)	2 mg 17-β-estradiol+ 1 mg norethisterone acetate or 2 mg 17-β-estradiol (hysterectomy)	NT	16 y	All-cause death, CVE,stroke,VT
Greenspan et al	2005	373	E/E + P:71.2(5.6) C:71.3(4.8)	0.625 mg conjugated equine estrogen+ 2.5 mg medroxyprogesterone or 0.625 mg conjugated equine estrogen (hysterectomy)	Placebo	3 y	All-cause death,VT
KEEPS	2014	727	E1 + P:52.8(2.6) E2 + P:52.7(2.6) C:52.5(2.5)	0.45 mg conjugated equine estrogen or 50 mcg transdermal 17-estradiol,each with 200 mg progesterone	Placebo	4 y	All-cause death, stroke,VT
Piret et al.^a^	2006	1778	E + P:58.5(3.9) C:59(3.9)	0.625 mg conjugated equine oestrogen,+ 2.5 mg medroxyprogesterone acetate, or 0.625 mg conjugated equine oestrogens+ 5 mg medroxyprogesterone acetate	Placebo	3.43 y	All-cause death, stroke
EPAT	2001	222	E:60.9(6.7) C:62.1(7.1)	1 mg micronized 17-β-estradiol	Placebo	2 y	stroke
WHI	2002	16,608	E + P:63.2(7.1) C:63.3(7.1)	0.625 mg conjugated equine estrogen+ 2.5 mg medroxyprogesterone acetate	Placebo	5.2 y	All-cause death, CVE,stroke,VT
WHI II	2004	10,739	E:63.6(7.3) C:63.6(7.3)	0.625 mg conjugated equine estrogen	Placebo	6.8 y	All-cause death, CVE,stroke,VT
HERS	1998	2763	E + P:67(7) C:67(7)	0.625 mg conjugated equine estrogen+ 2.5 mg medroxyprogesterone acetate	Placebo	4.1 y	All-cause death, CVE,stroke,VT
ESPIRT	2002	1017	E:62.3(5.2) C:62.9(4.9)	2 mg oestradiol valerate	Placebo	2 y	All-cause death, CVE,stroke,VT
WELL-HART	2003	226	E:61.8(6.7) E + P:64.4(6.4) C:64.2(6.2)	1 mg micronized 17-β-estradiol or 1 mg micronized 17-β-estradiol+ 5 mg of medroxyprogesterone acetate	Placebo	3.3 y	All-cause death
Catherine et al	2001	664	E:72(10) C:71(10)	1 mg 17-β-estradiol	Placebo	2.8 y	All-cause death, CVE,stroke,VT
ERT II	1979	168	E + P:55.3 C:54.9	2.5 mg conjugated estrogen+ 10 mg medroxyprogesterone acetate	Placebo	10 y	All-cause death, CVE
EVTET	2000	140	E + P:55.8(7) C:55.7(5.9)	2 mg estradiol+ 1 mg norethisterone acetate	Placebo	2 y	VT
STOP^b^	2001	489	E/E + P:72(4) C:71(4)	0.625 mg conjugated estrogen+ 2.5 mg medroxyprogesterone or 0.625 mg conjugated estrogen (hysterectomy)	Placebo	3 y	All-cause death, stroke,VT
WHISP	2006	100	E/E + P:69.4(8.6) C:68.3(9)	1 mg 17-β-estradiol+ 0.5 mg norethisterone acetate	Placebo	1 y	All-cause death, CVE,stroke,VT
WAVE	2002	423	E/E + P:65(9 C:66(9))	0.625 mg conjugated equine estrogen+ 2.5 mg medroxyprogesterone or 0.625 mg conjugated equine estrogen (hysterectomy)	Placebo	2.8 y	All-cause death, CVE,stroke,VT
WISDOM^c^	2007	6026	E + P:63.3(4.7) C:63.3(4.6)	0.625 mg conjugated equine estrogen+ 2.5 mg medroxyprogesterone	Placebo	10 y	All-cause death, CVE,VT
EAGAR	2006	83	E/E + P:64(8) C:64(9)	1 mg 17-β-estradiol + 2.5 mg medroxyprogesterone or 1 mg 17-β-estradiol (hysterectomy)	Placebo	3.5 y	CVE
J.E.B et al	2003	18	E:58.8(4.5) C:56.6(5)	50 μg estradiol	Placebo	4 w	FMD
Enderle et al	2000	20	E:64.9(7.2) C:64.9(7.2)	4 mg 17-β-estradiol	Placebo	2 h	FMD,NMD
Claire et al	2007	18	E + P:62(11) C:62(11)	10 μg ethinyl estradiol+ 1 mg norethisterone acetate	Placebo	3 m	FMD,NMD
Marie et al	1998	17	E:60(48–75) E + P: (48–75) C: (48–75)	0.2 mg estradiol or 0.2 mg estradiol+ 300 mg vaginal micronized progesterone	Placebo	14 w	FMD,NMD
Paola et al	2011	40	E + P:52(3.3) C:51.9(2.4)	1 mg estradiol+ 2 mg drospirenone	Placebo	6 m	FMD,NMD
Akihiko et al	2004	44	Elow:54.1(6.8) Ehigh:53.4(5.1) C:52.8(6.9)	0.625 mg conjugated equine estrogen or 0.3125 mg conjugated equine estrogen	NT	3 m	FMD,NMD
B.G et al	2001	51	E + P:55.1(5.3) C:55.4(6.4)	2 mg oestradiol+ 1 mg norethisterone acetate	Placebo	6 m	FMD,NMD
Antonino et al.^d^	2001	90	E + P:56(8) C:55(6)	1 mg 17-β-estradiol+ 0.5 mg norethisterone acetate	Placebo	6 m	FMD,NMD
Andrew et al	2007	100	CAD:67(8) H:65(7)	0.05 mg 17-β-estradiol or 0.05 mg 17-β-estradiol+ 0.14 mg norethisterone acetate	Placebo	18 h	FMD,NMD
W.Marchien et al	1999	27	E + P:52.1(0.9) C:53.2(0.9)	1 mg 17-β-estradiol+ 5 or 10 mg dydrogesterone for the duration of 12 months, 2 mg 17-β-estradiol+ 10 mg dydrogesterone for the final 3 months	Placebo	15 m	FMD,NMD
Mark K et al.^e^	2005	61	E + P:64(9) C:64(9)	0.625 mg conjugated equine estrogen+ 2.5 mg medroxyprogesterone acetate	Placebo	34 m	FMD,NMD
Kerrie L et al.^f^		36	Eoral:57(4) Etrans:57(4) C:56(7)	1 mg oral estradiol or 0.05 mg transdermal estradiol	Placebo	12 w	FMD
Aris et al	2012	84	E + P:48(4) C:50(3)	50 mcg 17-β-estradiol+ 200 mg micronized progesterone	NT	3 month	FMD,NMD
PERT	2020	172	E + P:51(3) C:51(3.2)	0.1 mg transdermal estradiol per day+ 200 mg intermittent micronized progesterone for 12 days per 2 month	Placebo	12 month	FMD,NMD

Abbreviations: *CVE* cardiovascular events, *VT* venous thromboembolism, *FMD* flow-mediated dilatation, *NMD* nitroglycerin-mediated dilation, *CAD* coronary heart disease

*H* health, *y* year, *h* hour, *m* month, *E* estrogen, *P* progesterone, *C* control, *NT* no treatment

Explanations: ^a^: treatment at blind HT and placebo selected for meta-analysis; ^b^: treatment at HRT/ERT and placebo selected for meta-analysis; ^c^: treatment at combined therapy and placebo selected for meta-analysis; ^d^: treatment at HRT and placebo selected for meta-analysis; ^e^: treatment at HRT intervention(placebo and active) selected for meta-analysis; ^f^: treatment at oral E2 and placebo selected for meta-analysis

### Risk of bias analysis

The overall bias risk of the included studies in this review was low. However, among them, ERT II (1979) had defects in randomization and allocation concealment, Aris (2012) and DOPS (2012) had defects in double-blinding and blind methods for outcome assessment, and WHISP (2006) had defects in incomplete outcome data. The above four studies were considered to have a high risk of bias [11, 21, 30, 36]. Risk assessment of study bias was summarized in Fig. S1. No strong evidence of publication bias was found. For the rating results of the GRADE method, PRISMA and MOOSE checklists can be found respectively in Table S1-S3.

### Main outcomes

#### All-cause death

**Fig. 2:**
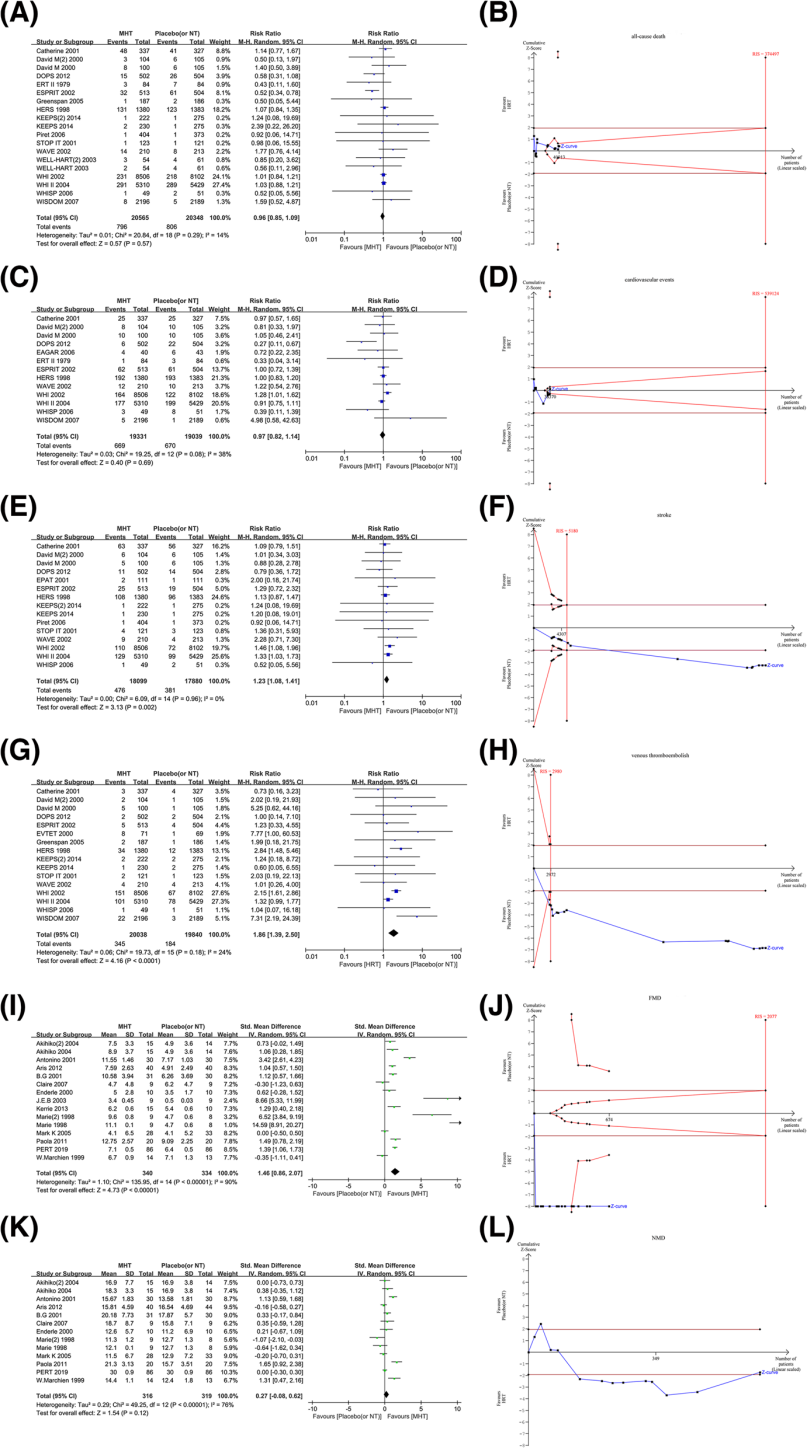
Different effects of MHT and placebo (or no treatment) on outcomes included in this review (**A-L**). Forest plot (**A**) and trial sequential analysis (**B**) of the effects of MHT and placebo (or no treatment) on all-cause death in postmenopausal women. Forest plot (**C**) and trial sequential analysis (**D**) of the effects of MHT and placebo (or no treatment) on cardiovascular events in postmenopausal women. Forest plot (**E**) and trial sequential analysis (**F**) of the effects of MHT and placebo (or no treatment) on stroke outcomes in postmenopausal women. Forest plot (**G**) and trial sequential analysis (**H**) of the effects of MHT and placebo (or no treatment) on venous thromboembolism in postmenopausal women. Forest plot (**I**) and trial sequential analysis (**J**) of the effects of MHT and placebo (or no treatment) on the improvement degree of FMD in postmenopausal women. Forest plot (**K**) and trial sequential analysis (**L**) of the effects of MHT and placebo (or no treatment) on the improvement degree of NMD in postmenopausal women

Nineteen studies reported all-cause death in 16 trials (*n* = 40,913) [8, 11, 19–26, 29, 30, 32–35]. There was no significant difference in the risk of all-cause death in the overall population of postmenopausal women receiving MHT compared with placebo (or no treatment) (796 vs 806; RR = 0.96, 95%CI 0.85 to 1.09, I^2^ = 14%; high-certainty evidence, Fig. 2A). TSA results of all-cause death showed that the cumulative Z-curve did not cross the conventional boundary and the trial sequential monitoring boundary, which confirmed the reliability of the negative conclusion drawn from meta-analysis, but the cumulative sample size did not reach the RIS to confirm this negative conclusion (RIS = 374,497, Fig. 2B).

#### Cardiovascular events

Thirteen studies reported cardiovascular events in 12 trials (*n* = 38,370) [8, 11, 22–24, 28, 32, 33, 35–38]. There was no significant difference in the risk of cardiovascular events between the overall population of postmenopausal women receiving MHT and placebo (or no treatment) (669 vs 670; RR = 0.97, 95%CI 0.82 to 1.14, I^2^ = 38%; high-certainty evidence, Fig. 2C). TSA results of cardiovascular events showed that the cumulative Z-curve did not cross the conventional boundary and the trial sequential monitoring boundary, which confirmed the reliability of the negative conclusion obtained from meta-analysis, but the cumulative sample size did not reach the RIS to confirm this negative conclusion (RIS = 539,124, Fig. 2D).

#### Stroke

**Fig. 3:**
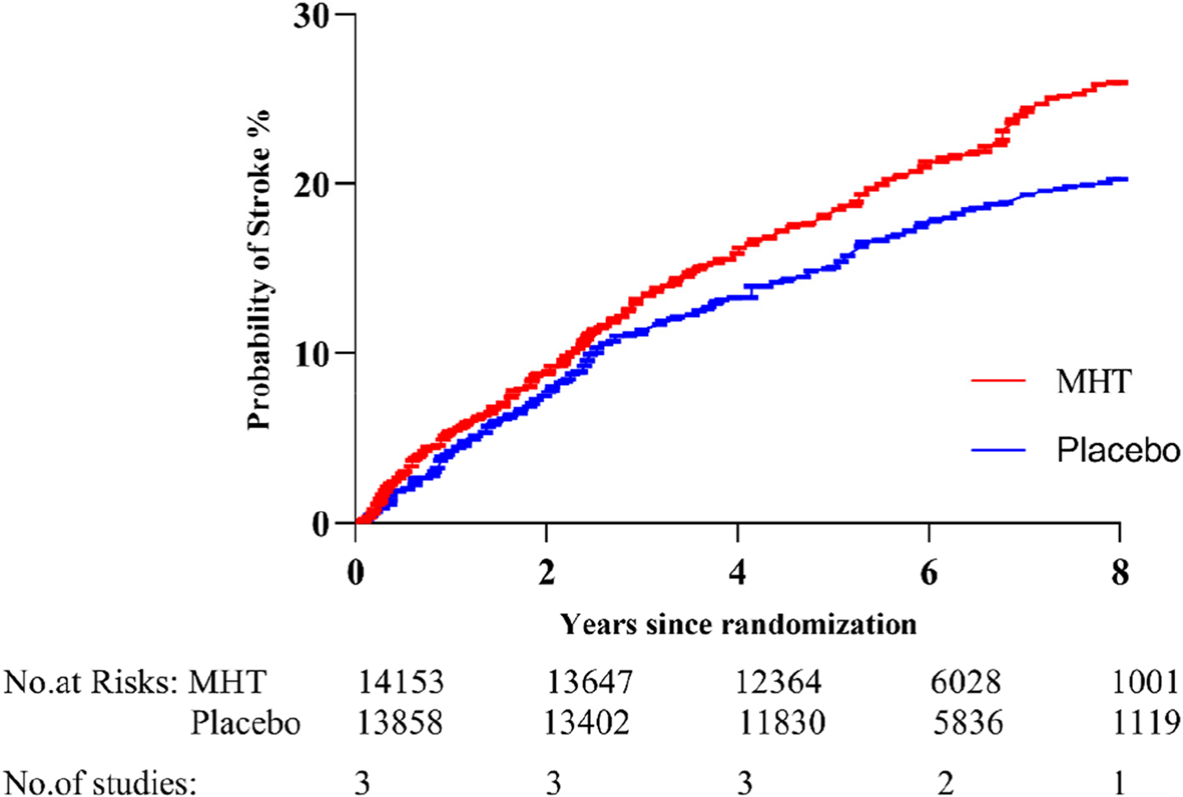
Kaplan Meier time survival curve of postmenopausal women receiving MHT and placebo for stroke outcomes. The red curve represented MHT, while the blue curve represented placebo treatment, and HR (1.31,95% CI [1.08 to 1.59]) was obtained by fitting the relationship between survival distributions and different interventions using the Cox regression model

Fifteen studies reported stroke outcome in 13 trials (*n* = 35,979) [8, 11, 19–22, 24, 25, 27, 29, 32, 34, 35]. Compared with placebo (or no treatment), MHT was significantly associated with the risk of stroke in the overall population of postmenopausal women (476 vs 381; RR = 1.23, 95%CI 1.08 to 1.41, I^2^ = 0%; high-certainty evidence, Fig. 2E). Three studies reported the onset time of stroke (14,153 subjects receiving MHT vs 13,858 subjects receiving placebo; HR = 1.31, 95%CI 1.08 to 1.59, Fig. 3). TSA results of stroke outcome showed that the cumulative Z-curve crossed the conventional boundary, and the cumulative sample size reached the RIS to confirm this positive conclusion (RIS = 5180, Fig. 2F), which confirmed the reliability of the positive conclusion obtained from meta-analysis.

#### Venous thromboembolism

Sixteen studies reported venous thromboembolism outcome in 14 trials (*n* = 39,878) [8, 11, 19–25, 28, 29, 33–35]. Compared with placebo (or no treatment), MHT was significantly related to the risk of venous thromboembolism in the overall population of postmenopausal women (345 vs 184; RR = 1.86, 95%CI 1.39 to 2.50, I^2^ = 24%; high-certainty evidence, Fig. 2G). TSA results of venous thromboembolism showed that the cumulative Z-curve crossed the conventional boundary and the trial sequential monitoring boundary, and the cumulative sample size reached the RIS to confirm this positive conclusion (RIS = 2980, Fig. 2H), which confirmed the reliability of the positive conclusion obtained from meta-analysis.

#### Flow-mediated arterial dilation(FMD)

Fifteen studies reported FMD outcome in 13 trials (*n* = 674) [36–45, 47–49]. Compared with placebo, MHT was significantly related to the improvement degree of FMD in the overall population of postmenopausal women (SMD = 1.46, 95%CI 0.86 to 2.07, I^2^ = 90%; moderate-certainty evidence, Fig. 2I). TSA results of FMD outcome showed that the cumulative Z-curve crossed the conventional boundary and the trial sequential monitoring boundary, which confirmed the reliability of the positive conclusions obtained from meta-analysis, although the Z-curve did not cross the RIS boundary (RIS = 2077, Fig. 2J).

#### Nitroglycerin-mediated arterial dilation(NMD)

Thirteen studies reported NMD outcome in 11 trials (*n* = 635) [36, 38–43, 45, 47–49]. There was no significant difference in the improvement degree of NMD between the overall postmenopausal women receiving MHT and placebo (or no treatment) (SMD = 0.27, 95%CI − 0.08 to 0.62, I^2^ = 76%; moderate-certainty evidence, Fig. 2K). TSA results of NMD outcome showed that the cumulative Z-curve did not cross the conventional boundary, which confirmed the reliability of the negative conclusion obtained from meta-analysis. However, the trial sequential monitoring boundary and RIS boundary were automatically ignored due to the small cumulative sample size (RIS = 15,737, Fig. 2L).

### Sensitivity analysis

FMD (I^2^ = 90%) and NMD (I^2^ = 76%) were considered to be highly heterogeneous in the outcomes of the above-included studies. Leave-one-out method was used for sensitivity analysis of studies related to FMD and NMD outcomes, and the amplitude of I^2^ change was not obvious (the average change value of FMD I^2^ = 0.53%, range 0 to 3%; the average change value of NMD I^2^ = 2.15%, range 0 to 9%). When we eliminated the studies with relatively large heterogeneity changes in these two outcomes (number of excluded trials: FMD *n* = 7, NMD *n* = 5), the I^2^ and SMD values of FMD and NMD outcomes changed to varying degrees, FMD (from: SMD = 1.46, 95%CI 0.86 to 2.07, I^2^ = 90% to SMD = 1.19, 95%CI 0.98 to 1.39, I^2^ = 0%, Fig. S2A), NMD (from: SMD = 0.27, 95%CI − 0.08 to 0.62, I^2^ = 76% to SMD = 0.03, 95%CI − 0.15 to 0.22, I^2^ = 0%, Fig. S2B), but the conclusions obtained through meta-analysis had not changed. The funnel plot of FMD showed that there were two studies (J.E.B 2003 and Marie (2) 1998) with significant deviation compared with the other 13 studies, but the conclusions of these two studies were positive, and one of them (Marie (2) 1998) was a part of Marie 1998 trial, we considered publication bias for FMD to be at low risk (Fig. S3A and B). Apart from that, we found that the duration of treatment in the studies related to FMD and NMD outcomes was significantly different (range 2 hours to 34 months). To assess the impact of this, we conducted a subgroup analysis of different treatment duration ranges (< 1 month, ≥1 month and < 6 months, ≥ 6 months and < 12 months, ≥ 12 months). In the two subgroups of treatment time range < 1 month (SMD = 4.47, 95%CI − 3.40 to 12.35, I^2^ = 95%) and ≥ 12 months (SMD = 0.38, 95%CI − 0.77 to 1.53, I^2^ = 93%), there was no significant difference between MHT and placebo (or no treatment) in the improvement degree of FMD, which changed the positive conclusion obtained from meta-analysis. However, due to fewer included studies (< 1 month: 2 studies; ≥ 12 months: 3 studies) and large heterogeneity, the reliability of this conclusion was low (Fig. S4A). In the subgroup of NMD outcome ≥6 months and < 12 months, MHT was significantly correlated with the improvement of NMD (SMD = 1.01, 95%CI 0.26 to 1.75, I^2^ = 79%), which changed the negative conclusion obtained from meta-analysis. Similarly, due to fewer included studies (≥ 6 months and < 12 months: 3 studies) and large heterogeneity, the reliability of the conclusion was not high (Fig. S4B).

### Subgroup analysis

#### The onset time of MHT

To assess the effects of MHT onset time and “time hypothesis”, we stratified trials for each outcome. The specific stratification criterion was < 10 years or ≥ 10 years since the onset of MHT after menopause, and if this stratified criterion was limited, the mean age of subjects at baseline would be used as a substitute. To minimize the limitations of using baseline age to replace MHT onset time stratification as much as possible, and highlight the characteristic differences between subgroups, we selected the subjects with mean age at baseline ≤60 years or ≥ 65 years as the stratified conditions for all-cause death, cardiovascular events, stroke, and venous thromboembolism, and the average age ≤ 59 years or ≥ 64 years as stratified conditions for FMD and NMD. The results of the subgroup analysis provided some evidence for the “time hypothesis” of all-cause death, cardiovascular events, and improvement in FMD. Compared with women started MHT more than 10 years after menopause, women started MHT within 10 years after menopause had lower frequency of all-cause death and cardiovascular events, and more significant improvement in FMD (all-cause death *P* = 0.02, cardiovascular events *P* = 0.002, FMD *P* = 0.0003, Fig. S5A, B and E), while women started MHT more than 10 years after menopause did not improve. In addition, we found that the improvement of NMD in women started MHT within 10 years after menopause seemed to be better than that in women started MHT more than 10 years after menopause (NMD *P* = 0.08, Fig. S5F), while stroke (*P* = 0.53, Fig. S5C), venous thromboembolism (*P* = 0.79, Fig. S5D) did not show significant risk difference between subgroups.

#### Primary prevention and secondary prevention

The above conclusions indicated that MHT could not reduce the risk of cardiovascular disease in the overall population of postmenopausal women. We stratified the subjects according to their baseline health status (healthy postmenopausal women or postmenopausal women with coronary heart disease and atherosclerotic vascular disease) to assess the effects of MHT on the cardiovascular system as an intervention of different prevention levels (primary or secondary prevention) of cardiovascular diseases. There was no evidence of statistically significant difference between the subgroups of primary and secondary prevention for the six outcomes included in this systematic review (Fig. S6A-F). It was noteworthy that the improvement of FMD in postmenopausal women with coronary heart disease and atherosclerotic vascular disease appears to be more significant compared with healthy postmenopausal women (FMD *P* = 0.08, Fig. S6E), although the statistical requirements were not met, this might be due to the cumulative sample size not reaching the RIS, which was worthy of further study in the future.

#### Treatment protocol

Progesterone has been believed to have the effect of antagonizing estrogen. To evaluate the effects of different treatment protocols (mono-estrogen therapy or combination therapy of estrogen and progesterone) on the outcomes of the included study, we conducted a subgroup analysis of this, but there was no evidence to suggest that the six outcomes included in this systematic review were statistically different between the subgroups of mono-estrogen therapy or combination therapy of estrogen and progesterone (Fig. S7A-F).

## Discussion

### Main findings

This systematic review and meta-analysis of 33 RCTs included a total of 44,639 postmenopausal women (average age from 48 to 72), who received MHT (mono-estrogen therapy or combination therapy of estrogen and progesterone). MHT fails to reduce the risk of all-cause death, and cardiovascular events, but increases the risk of stroke (the stroke events observed in relevant trials using MHT were predominantly constituted by ischaemic stroke rather than hemorrhagic stroke [50, 51]) and venous thromboembolism in postmenopausal women. Although our analysis suggests that MHT could improve arterial dilation in postmenopausal women, moreover, a formal study-level meta-analysis conducted by Casanova G et al. showed that MHT could reduce the concentrations of low-density lipoprotein cholesterol (LDL-C) and triglyceride (TG) in serum [52], which was clearly contradictory to the therapeutic guideline that every 1% reduction in LDL-C levels can reduce the risk of atherosclerotic cardiovascular disease (ASCVD) by about 1% [53]. The significantly increased risk of cardiovascular events in the overall population of postmenopausal women compared with premenopausal women also suggests that estrogen has a certain degree of protection against the cardiovascular system.

**Fig. 4:**
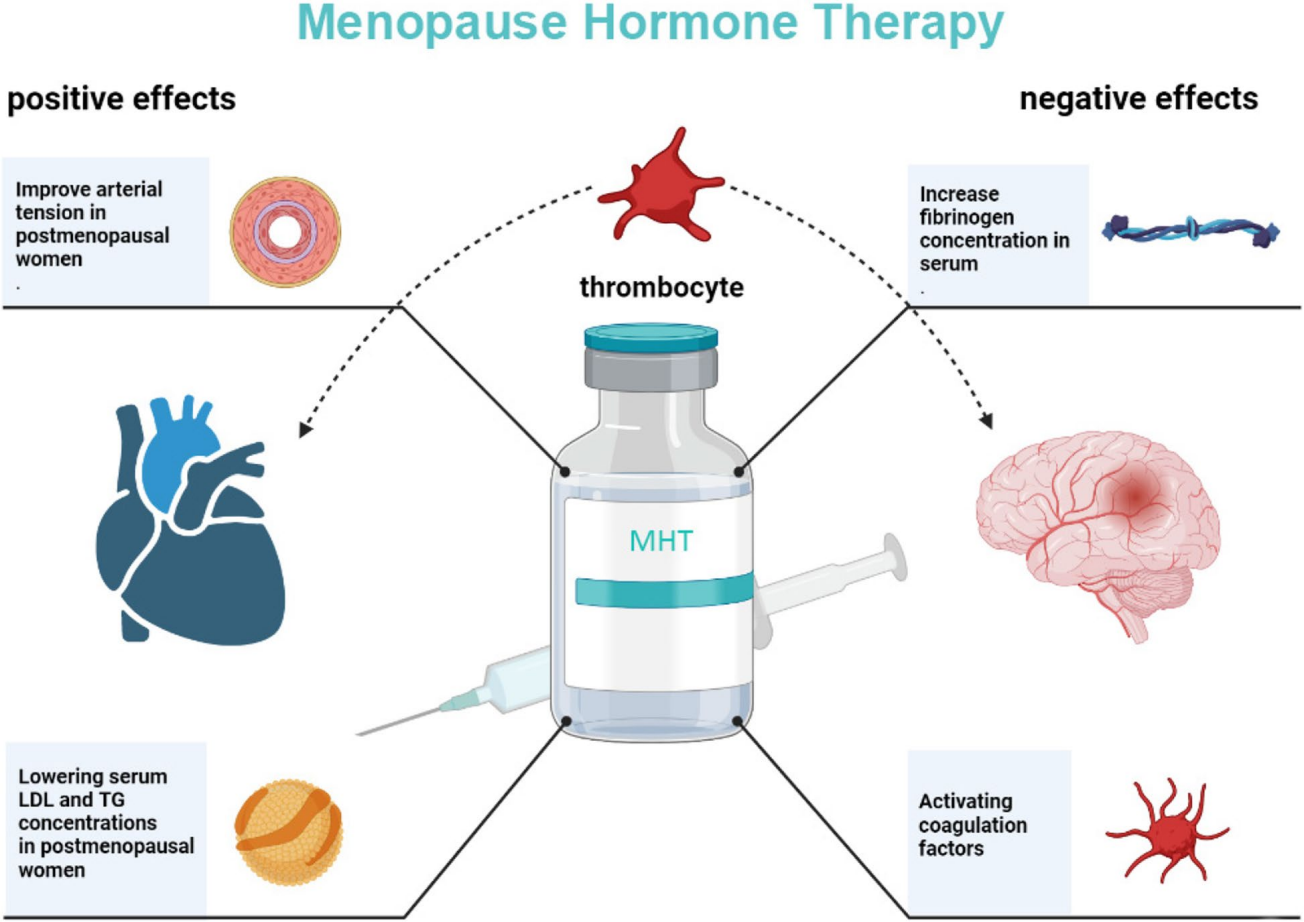
Positive effects and negative effects of MHT on postmenopausal women. MHT can induce stroke and cardiovascular events by increasing the concentration of fibrinogen in serum and activating coagulation factors, and partially counteract the positive effects of MHT (improvement of arterial vascular tension and concentrations of LDL and TG in serum), which may be one of the reasons for the above contradictory results

Subgroup analysis of MHT onset time showed that menopausal women received MHT in the early period (within 10 years after menopause or age ≤ 60 years old) had significantly better benefits on all-cause death, cardiovascular events, and arterial dilation than those received MHT in the late period (more than 10 years after menopause or age ≥ 65 years old), suggesting that the increased age-related risk of cardiovascular disease might be responsible for the above conflicting results. It should be noticed that we might have focused too much on the effects of MHT on different outcomes and overlooked the linkages between them. As for stroke, venous thromboembolism, and cardiovascular event, their main pathogenic factors are similar: thrombosis and blockage of blood vessels leading to the loss of vital organ function [54, 55]. Results from clinical trials had shown that mean platelet volume could independently predict the prognosis of patients with acute myocardial infarction, including death [56, 57]. Mean platelet volume is highly correlated with the risk of venous thromboembolism [58]. Estrogen could increase the concentration of fibrinogen in serum and activate coagulation factors, leaving the blood in a hypercoagulable state [59]. This means that MHT could further induce stroke and cardiovascular events by promoting blood to be in a hypercoagulable state, and partially offset the positive effects of MHT, which might be another potential cause of the above contradictory results (Fig. 4). However, we did not find long-term, large sample clinical trials targeting at the effects of MHT combined with antiplatelet drugs on cardiovascular risk in postmenopausal women.

In addition, in the subgroup analysis of primary prevention and secondary prevention, the improvement of FMD in menopausal women with coronary heart disease and atherosclerotic vascular disease seemed to be more obvious than that in healthy menopausal women, but more relevant clinical trials were needed to support this conclusion.

The addition of progesterone in MHT did not alter the effect of estrogen on postmenopausal women’s all-cause death, cardiovascular events, stroke, venous thromboembolism, and arterial dilation.

The subgroup analysis results of the influence of different treatment duration on the improvement of FMD and NMD in postmenopausal women receiving MHT and placebo (or no treatment) showed that the effect of MHT on FMD in postmenopausal women might not be apparent until a certain duration of treatment (≥ 1 month) was reached, however, an excessively prolonged duration of treatment (≥ 12 months) might weaken the effect of MHT. The optimal duration of MHT for NMD in postmenopausal women was similar to that of FMD (≥ 6 months and < 12 months), due to the limitation of sample size and heterogeneity, the reliability of subgroup analysis conclusion of the duration of treatment was not high, which needed further researches to verify.

Although the credibility of evidence in this systematic review was relatively stable, the outcomes of the included studies, except for stroke, venous thromboembolism, and FMD, the cumulative sample size involved in other outcome-related trials did not reach the RIS to prove the conclusion. Therefore, more clinical trials that meet the inclusion criteria would be needed to provide data support in the future to further improve the quality of evidence.

### Strengths and limitations

One of the strengths of our study was that the included studies were randomized controlled trials with a low overall risk of bias rather than open-label or observational studies, and the large sample sizes of the included studies ensured more precise analysis of outcomes. However, this review has the following limitations:

First, when we verified the “time hypothesis” through subgroup analysis of treatment onset time, some trials used the average age of subjects at baseline as the stratified condition due to the limitation of research data. Although we believed that most subjects were correctly allocated, it was inevitable that a small number of subjects might be misallocated.

Second, due to the complexity of drug types and space limitation of the article, we did not conduct a complete subgroup analysis on different doses, administration routes, and treatment duration of MHT.

Third, the studies of FMD and NMD outcomes were highly heterogeneous. Although we had proved the reliability of the conclusions by multiple methods such as single exclusion, partial exclusion, and subgroup analysis, we could not completely ignore its impact on the conclusions.

Fourth, meta-analysis and trial sequential analysis methods were used for multiple verifications of conclusions in this systematic review, but this might increase the risk of type I error.

Fifth, the cumulative sample size involved in trials related to all-cause death, cardiovascular events, and NMD outcomes did not reach the RIS to support the conclusions, and more clinical trials that meet the inclusion criteria were needed to provide data support.

## Conclusions

MHT could improve flow-mediated arterial dilation (FMD) in the overall population of postmenopausal women, but fails to lower the risk of all-cause death and cardiovascular events. Moreover, it woud increase the risk of stroke and venous thrombosis. Women who received MHT within 10 years after menopause would benefit more from the improved FMD and the reduced risk of all-cause mortality and cardiovascular events, although the risk of stroke and venous thrombosis is not reduced. There is no difference in the outcome of cardiovascular system endpoints between the two protocols (mono-estrogen therapy and combination therapy of estrogen and progesterone).

### Supplementary Information

**Additional file 1: Methods S1.** Search strategies. **Table S1.** Evaluation of evidence quality based on GRADE approach. **Table S2.** PRISMA checklist Table S3 MOOSE checklist. **Figure S1.** Evaluate the bias risk of trials included in the systematic review based on the bias risk assessment criteria in Cochrane. Handbook for Systematic Reviews of Interventions. **Figure S2.** Sensitivity Analysis (leave one-out method). **Figure S3.** Funnel plots. **Figure S4 .**Subgroup analysis of different MHT treatment durations. **Figure S5.** Subgroup analysis of MHT onset time. **Figure S6.** Subgroup analysis of primary prevention and secondary prevention of MHT. **Figure S7.** Subgroup analysis of MHT protocols.

## Data Availability

All data generated or analyzed during this study are included in this published article and its supplementary information files. Besides, all data are available from the first author on reasonable request.

## Abbreviations

MHT: Menopause hormone therapy
FMD: Flow-mediated arterial dilation
NMD: Nitroglycerin-mediated arterial dilation
ASCVD: Atherosclerotic cardiovascular disease
LDL-C: Low-density lipoprotein cholesterol
TG: Triglyceride
RCTs: Randomized controlled trials
RR: Risk ratio
HR: Hazard ratio
CI: Confidence interval
SMD: Standardized mean difference
RIS: Required information size
