# Supplementary material for: The benefits and risks of menopause hormone therapy for the cardiovascular system in postmenopausal women: a systematic review and meta-analysis
Source: BMC Womens Health. 2024 Jan 23;24:60. doi: 10.1186/s12905-023-02788-0 (PMC10804786; doi:10.1186/s12905-023-02788-0)
Supplement: Supplementary file 1 — Additional file 1: Methods S1. Search strategies. Table S1. Evaluation of evidence quality based on GRADE approach. Table S2. PRISMA checklist Table S3 MOOSE checklist. Figure S1. Evaluate the bias risk of trials included in the systematic review based on the bias risk assessment criteria in Cochrane. Handbook for Systematic Reviews of Interventions. Figure S2. Sensitivity Analysis (leave one-out method). Figure S3. Funnel plots. Figure S4 .Subgroup analysis of different MHT treatment durations. Figure S5. Subgroup analysis of MHT onset time. Figure S6. Subgroup analysis of primary prevention and secondary prevention of MHT. Figure S7. Subgroup analysis of MHT protocols. [file 12905_2023_2788_MOESM1_ESM.pdf]

Supplementary Materials

The Benefits and Risks of Menopause Hormone Therapy for the Cardiovascular System in Postmenopausal Women: A Systematic Review and Meta-analysis

Methods S1 **Search strategies**

Table S1 **Evaluation of evidence quality based on GRADE approach**

Table S2 **PRISMA checklist**

Table S3 **MOOSE checklist**

Figure S1 **Evaluate the bias risk of trials included in the systematic review based on the bias risk assessment criteria in *Cochrane Handbook for Systematic Reviews of Interventions***

Figure S2 **Sensitivity Analysis (leave one-out method)**

Figure S3 **Funnel plots**

Figure S4 **Subgroup analysis of different MHT treatment durations**

Figure S5 **Subgroup analysis of MHT onset time**

Figure S6 **Subgroup analysis of primary prevention and secondary prevention of MHT**

Figure S7 **Subgroup analysis of MHT protocols**

Methods S1　Search strategies(search date 15/07/2022)

Cochrane Controlled Trial Register (search date 15/07/2022)
**Search Strategies**
1 Mesh descriptor (estradiol) explode all trees
2 Mesh descriptor (hormone replacement therapy) explode all trees
3 menopause hormone therapy
4 hot flashes
5 menopause* or postmenopause*
6 cardiovascular
7 stroke*
8 Mesh descriptor (thromosis and embolish) explode all trees
9 cardiovascular death
10 MHT or HT or ORT or ERT or HRT
11 estrogen or oestrogen
12 hyperlipidemia*
13 (1 or 2 or 3 or 4 or 5 or 6 or 7 or 8 or 9 or 10 or 11 or 12)
14 Mesh descriptor (endothelium) explode all trees
15 vascular endothelium dependent relaxation
16 vasorelaxation* or vasodilatation* or vasodilation*
17 flow mediate
18 nitroglycerin* or glyceryl trinitrate* or trinitrate*
19 endothelium function
20 FMD or NMD
21 (14 or 15 or 16 or 17 or 18 or 19 or 20)
22 nitrate medidate
23 endothelial*
24 brachial artery or arteria brachialis
25 vascular effect

Cochrane Controlled Trial Register (search date 15/07/2022)
**Search Strategies**
26 brachial artery diameter
27 (22 or 23 or 24 or 25 or 26)
28 (13 and 21)
29 (13 and 27)

MEDLINE
**Search Strategies**
1 exp estradiol/
2 exp hormone replacement therapy /
3 estrogen$.mp.
4 exp menopause hormone therapy/
5 hot flashes.mp.
6 exp menopause/
7 cardiovascular.mp.
8 stroke$.mp.
9 exp thromosis/ or thromosis.mp.
10 exp embolish / or embolish.mp.
11 hyperlipidemia.mp.
12 (1 or 2 or 3 or 4 or 5 or 6 or 7 or 8 or 9 or 10 or 11)
13 exp endothelium/
14 vascular endothelium dependent.mp.
15 flow mediate.mp.
16 endothelium function.mp.
17 brachial.mp. or exp brachial artery/
18 endothelial$.mp.
19 (13 or 14 or 15 or 16 or 17 or 18)

MEDLINE
**Search Strategies**
20 (12 and 19)

EMBASE
**Search Strategies**
1 exp estradiol/
2 exp hormone replacement therapy /
3 estrogen.mp. or exp estrogen/
4 hot flashes.mp.
5 exp menopause/ or postmenopause.mp.
6 cardiovascular.mp.
7 stroke.mp.
8 exp thromosis/ or thromosis.mp.
9 exp embolish / or embolish.mp.
10 exp menopause hormone therapy/
11 exp hyperlipidemia/
12 cardiovascular death.mp.
13 (1 or 2 or 3 or 4 or 5 or 6 or 7 or 8 or 9 or 10 or 11 or 12)
14 exp endothelium/
15 nitrate medidate.mp.
16 flow mediate.mp.
17 exp endothelium function/
18 brachial.mp. or exp brachial artery/
19 endothelial.mp.
20 (14 or 15 or 16 or 17 or 18 or 19)
21 (13 and 20)

Table S1 Evaluation of evidence quality based on GRADE approach

Certainty assessment							№ of patients		Effect		Certainty	Importance
№ of studies	Study design	Risk of bias	Inconsistency	Indirectness	Imprecision	Other considerations	Menopause hormone therapy	Placebo(or NT)	Relative (95% CI)	Absolute (95% CI)		
**Death(all causes)**												
19	randomised trials	not serious	not serious	not serious	not serious	none	796/20565 (3.9%)	806/20348 (4.0%)	**RR 0.96** (0.85 to 1.09)	**2 fewer per 1,000** (from 6 fewer to 4 more)	⊕⊕⊕⊕ High	Important(5)
**Cardiovascular events**												
13	randomised trials	not serious	not serious	not serious	not serious	none	669/19331 (3.5%)	670/19039 (3.5%)	**RR 0.97** (0.82 to 1.14)	**1 fewer per 1,000** (from 6 fewer to 5 more)	⊕⊕⊕⊕ High	Important(5)
**Stroke**												
15	randomised trials	not serious	not serious	not serious	not serious	strong association	476/18099 (2.6%)	381/17880 (2.1%)	**RR 1.23** (1.08 to 1.41)	**5 more per 1,000** (from 1 more to 9 more)	⊕⊕⊕⊕ High	Critical(8)
**Venous thrombosis**												
16	randomised trials	not serious	not serious	not serious	not serious	strong association	345/20038 (1.7%)	184/19840 (0.9%)	**RR 1.86** (1.39 to 2.50)	**8 more per 1,000** (from 4 more to 14 more)	⊕⊕⊕⊕ High	Critical(8)
**Flow-mediated vasodilation**												
15	randomised trials	not serious	very serious[a]	not serious	not serious	strong association	340	334		SMD **1.46 higher** (0.86 higher to 2.07 higher)	⊕⊕⊕◯ Moderate	Important(4)
**Nitroglycerin mediated vasodilation**												
13	randomised trials	not serious	serious[b]	not serious	not serious	none	316	319		SMD **0.27 higher** (0.08 lower to 0.62 higher)	⊕⊕⊕◯ Moderate	Important(4)

CI: confidence interval; **RR:** risk ratio; **SMD:** standardized mean difference.

Explanations

a. Could be imprecise due to I2=90%, Although a large number of clinical trials have proved its strong correlation.Nevertheless,we conservatively rated down for imprecision.

b. Could be imprecise due to I2=76%, we conservatively rated down for imprecision

Table S2 PRISMA checklist

Section and Topic	Item #	Checklist item	Location where item is reported
**TITLE**			
Title	1	Identify the report as a systematic review and meta analysis.	1
**ABSTRACT**			
Structure summary	2	Provide a structured summary including, as applicable: background; objectives; data sources; study eligibility criteria, participants, and interventions; study appraisal and synthesis methods; results; conclusions and implications of key findings.	2-3
**INTRODUCTION**			
Rationale	3	Describe the rationale for the review in the context of existing knowledge.	2-3
Objectives	4	Provide an explicit statement of the subject(s), intervention(s),outcome(s) and study design the review addresses.	4-5
**METHODS**			
Protocol and registration		Indicate if a review protocol exists, if and where it can be accessed, if available, provide registration information such as registration number.	5-6
Eligibility criteria	5	Specify the inclusion and exclusion criteria for the review and how studies were grouped for the syntheses.	6
Information sources	6	Specify all databases, registers, websites, organisations, reference lists and other sources searched or consulted to identify studies. Specify the date when each source was last searched or consulted.	6
Search strategy	7	Present the full search strategies for all databases, registers and websites, including any filters and limits used.	sMethods.1
Selection process	8	Specify the methods used to decide whether a study met the inclusion criteria of the review, including how many reviewers screened each record and each report retrieved, whether they worked independently, and if applicable, details of automation tools used in the process.	6
Data collection process	9	Specify the methods used to collect data from reports, including how many reviewers collected data from each report, whether they worked independently, any processes for obtaining or confirming data from study investigators, and if applicable, details of automation tools used in the process.	6-7

Section and Topic	Item #	Checklist item	Location where item is reported
Data items	10	List and define all other variables for which data were sought (e.g. participant and intervention characteristics). Describe any assumptions made about any missing or unclear information.	6-7
Study risk of bias assessment	11	Specify the methods used to assess risk of bias in the included studies, including details of the tool(s) used, how many reviewers assessed each study and whether they worked independently, and if applicable, details of automation tools used in the process.	8
Synthesis methods	13	Describe any methods required to prepare the data for presentation or synthesis, such as handling of missing summary statistics, or data conversions.	8
Reporting bias assessment	14	Describe any methods used to assess risk of bias due to missing results in a synthesis (arising from reporting biases).	8
Certainty assessment	15	Describe any methods used to assess certainty (or confidence) in the body of evidence for an outcome.	8
**RESULTS**			
Study selection	16	Describe the results of the search and selection process, from the number of records identified in the search to the number of studies included in the review, ideally using a flow diagram.	9,Figure1
Study characteristics	17	Cite each included study and present its characteristics.	9,Table1
Risk of bias in studies	18	Present assessments of risk of bias for each included study.	10,sFigure1
Results of individual studies	19	For all outcomes, present, for each study: (a) summary statistics for each group (where appropriate) and (b) an effect estimate and its precision (e.g. confidence/credible interval), ideally using structured tables or plots.	10-14, Figure2(A-L),Figure3
Results of syntheses	20	Present results of all investigations of possible causes of heterogeneity among study results.	14-15, sFigure2,4
Reporting biases	21	Present assessments of risk of bias due to missing results (arising from reporting biases) for each synthesis assessed.	14-15, sFigure1,3
Certainty of evidence	22	Present assessments of certainty (or confidence) in the body of evidence for each outcome assessed.	eTable1
Additional analysis		Give results of additional analysis, if done (e.g., sensitivity or subgroup analysis, meta-regression	15-17, sFigure5-7
**DISCUSSION**			

Section and Topic	Item #	Checklist item	Location where item is reported
Discussion	23	Provide a general interpretation of the results in the context of other evidence.	17-20, Figure4
Limitations	24	Discuss any limitations of the evidence included in the review.	20-21
**OTHER INFORMATION**			
Support	25	Describe sources of financial or non-financial support for the review, and the role of the funders or sponsors in the review.	21-22
Competing interests	26	Declare any competing interests of review authors.	22

Table S3 MOOSE checklist

Recommendation	Reported on Page No
**Reporting of background should include**	
Problem definition	Background (Page 2)
Hypothesis statement	Introduction (Page 4-5)
Description of study outcome(s)	All-cause death, cardiovascular events, stroke, venous thromboembolism, FMD, NMD
Type of exposure or intervention used	MHT
Type of study designs used	Systematic review and Meta-analysis
Study population	Postmenopausal women
**Reporting of search strategy should include**	
Qualifications of searchers (eg, librarians and investigators)	Investigator (Page 6)
Search strategy, including time period included in the synthesis and key words	Eligibility criteria, information sources, search strategy (Page 6) and Methods S1
Effort to include all available studies, such as digitize and extract data if data were only presented as graphs	GetData Graph Digitizer 2.24 was applied to digitize and extract the data
Databases and registries searched	Eligibility criteria, information sources, search strategy (Page 6)
Search software used, name and version, including special features used (eg, explosion)	Review Manager (RevMan5.4.1)<br>GetDataGraph Digitizer 2.24<br>TSA-0.9.5.10-Beta
Use of hand searching (eg, reference lists of obtained articles)	Eligibility criteria, information sources, search strategy (Page 6)

Recommendation	Reported on Page No
List of citations located and those excluded, including justification	Flow diagram in Figure 1
Method of addressing articles published in languages other than English	Eligibility criteria, information sources, search strategy (Page 6)
Method of handling abstracts and unpublished studies	Methods (Page 6)
Description of any method to extract data (if data were only presented as graphs)	Methods (Page 6-7)
**Reporting of methods should include**	
Description of relevance or appropriateness of studies assembled for assessing the hypothesis to be tested	Methods (Page 6-7)
Rationale for the selection and coding of data (eg, sound clinical principles or convenience)	Methods (Page 6-7)
Documentation of how data were classified and coded (eg, multiple raters, blinding and interrater reliability)	Methods (Page 6-7)
Assessment of confounding (eg, comparability of cases and controls in studies where appropriate)	Methods (Page 7)
Assessment of study quality, including blinding of quality assessors, stratification or regression on possible predictors of study results	Methods (Page 8)
Assessment of heterogeneity	Methods (Page 8)
Description of statistical methods (eg, complete description of fixed or random effects models, justification of whether the chosen models account for predictors of study results, dose-response models, or cumulative meta-analysis) in sufficient detail to be replicated	Methods (Page 7)
Provision of appropriate tables and graphics	Additional materials and supplementary materials

Recommendation	Reported on Page No
**Reporting of results should include**	
Graphic summarizing individual study estimates and overall estimate	Figure 2A-2L, Figure 3
Table giving descriptive information for each study included	Table 1
Results of sensitivity testing (eg, subgroup analysis)	Sensitivity analysis (Page 14-15)
Indication of statistical uncertainty of findings	Discussion (Page 19)
**Reporting of discussion should include**	
Quantitative assessment of bias (eg, publication bias)	Discussion (Page 20)
Justification for exclusion (eg, exclusion of non-English language citations)	Discussion (Page 20)
Assessment of quality of included studies	Discussion (Page 20)
**Reporting of conclusions should include**	
Consideration of alternative explanations for observed results	Conclusions (Page 21)
Generalisation of the conclusions (ie, appropriate for the data presented and within the domain of the literature review)	Conclusions (Page 21)
Guidelines for future research	Discussion (Page 20)
Disclosure of funding source	Grant Support (Page 22)

Figure S1 Evaluate the bias risk of trials included in the systematic review based on the bias risk assessment criteria in *Cochrane Handbook for Systematic Reviews of Interventions*

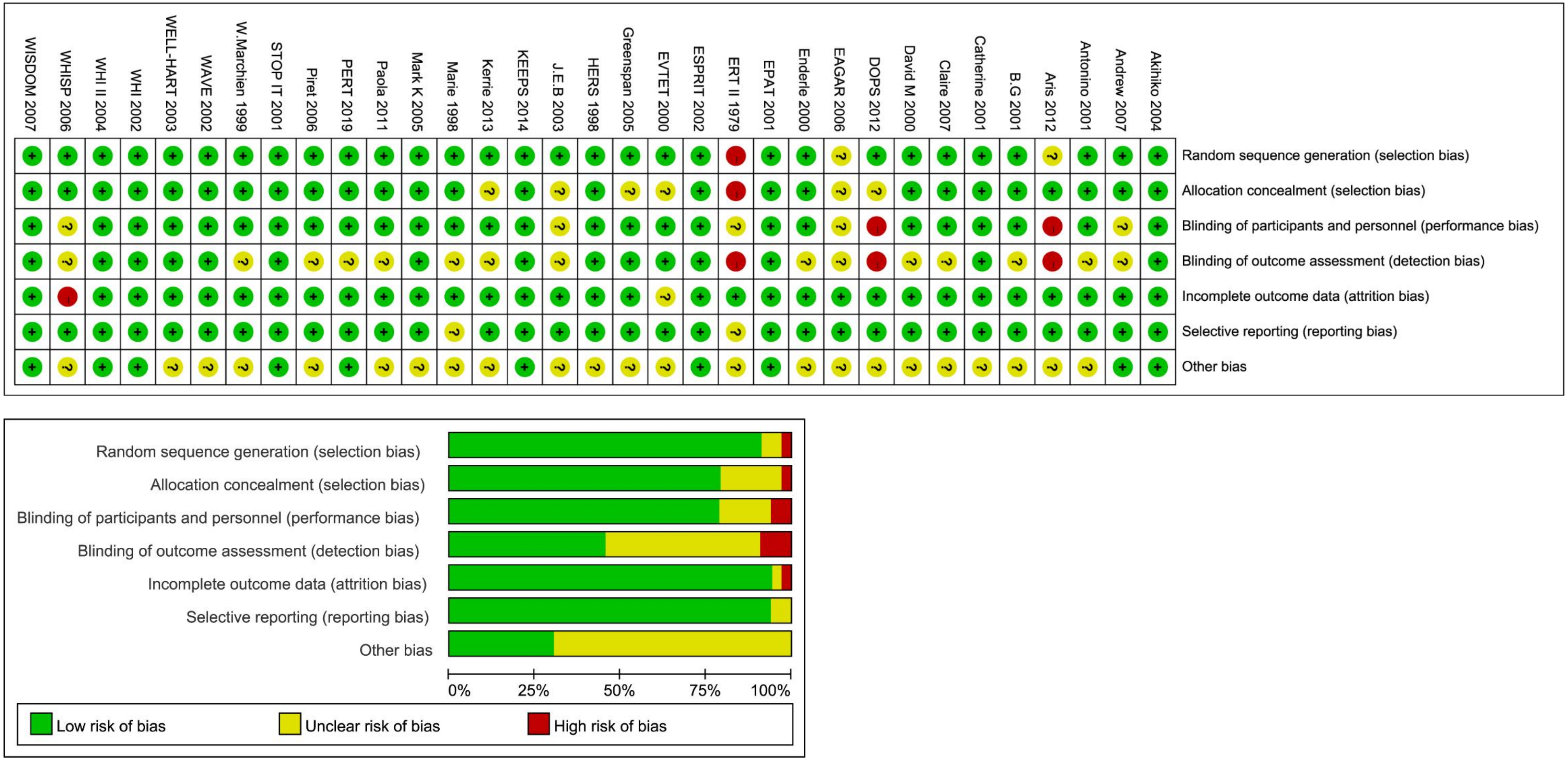

Figure S2 Sensitivity Analysis (leave one-out method)

(A)

Study or Subgroup	MHT Mean	MHT SD	MHT Total	Placebo(or NT) Mean	Placebo(or NT) SD	Placebo(or NT) Total	Weight	Std. Mean Difference IV, Random, 95% CI
Akihiko(2) 2004	7.5	3.3	15	4.9	3.6	14	7.1%	0.73 [-0.02, 1.49]
Akihiko 2004	8.9	3.7	15	4.9	3.6	14	6.5%	1.06 [0.28, 1.85]
Antonino 2001	11.55	1.46	30	7.17	1.03	30	0.0%	3.42 [2.61, 4.23]
Aris 2012	7.59	2.63	40	4.91	2.49	40	18.4%	1.04 [0.57, 1.50]
B.G 2001	10.58	3.94	31	6.26	3.69	30	13.7%	1.12 [0.57, 1.66]
Claire 2007	4.7	4.8	9	6.2	4.7	9	0.0%	-0.30 [-1.23, 0.63]
Enderle 2000	5	2.8	10	3.5	1.7	10	5.0%	0.62 [-0.28, 1.52]
J.E.B 2003	3.4	0.45	9	0.5	0.03	9	0.0%	8.66 [5.33, 11.99]
Kerrie 2013	6.2	0.6	15	5.4	0.6	10	5.1%	1.29 [0.40, 2.18]
Marie(2) 1998	9.6	0.8	9	4.7	0.6	8	0.0%	6.52 [3.84, 9.19]
Marie 1998	11.1	0.1	9	4.7	0.6	8	0.0%	14.59 [8.91, 20.27]
Mark K 2005	4.1	6.5	28	4.1	5.2	33	0.0%	0.00 [-0.50, 0.50]
Paola 2011	12.75	2.57	20	9.09	2.25	20	8.0%	1.49 [0.78, 2.19]
PERT 2019	7.1	0.5	86	6.4	0.5	86	36.2%	1.39 [1.06, 1.73]
W.Marchien 1999	6.7	0.9	14	7.1	1.3	13	0.0%	-0.35 [-1.11, 0.41]
**Total (95% CI)**			**232**			**224**	**100.0%**	**1.19 [0.98, 1.39]**

Heterogeneity: Tau² = 0.00; Chi² = 5.66, df = 7 (P = 0.58); I² = 0%
Test for overall effect: Z = 11.57 (P < 0.00001)

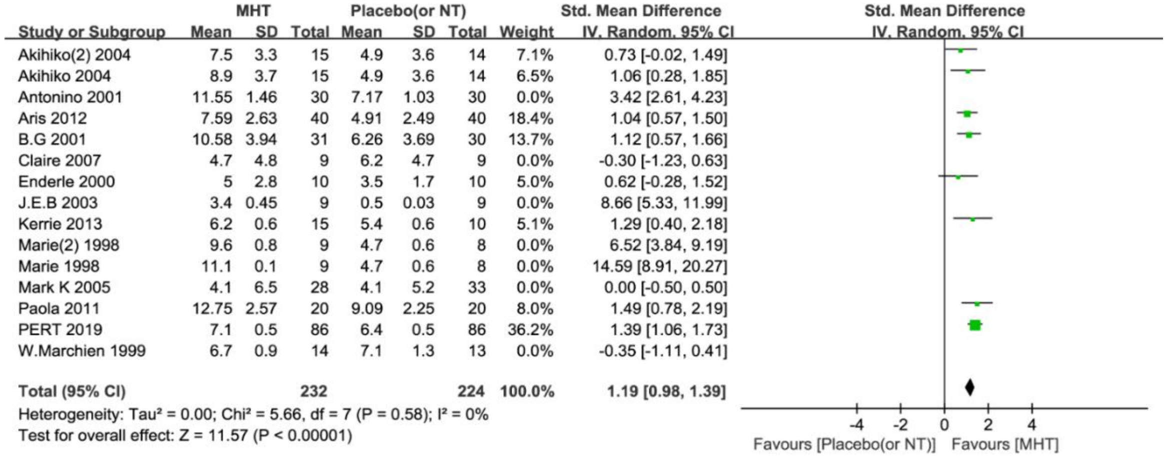

(B)

Study or Subgroup	MHT Mean	MHT SD	MHT Total	Placebo(or NT) Mean	Placebo(or NT) SD	Placebo(or NT) Total	Weight	Std. Mean Difference IV, Random, 95% CI
Akihiko(2) 2004	16.9	7.7	15	16.9	3.8	14	6.2%	0.00 [-0.73, 0.73]
Akihiko 2004	18.3	3.3	15	16.9	3.8	14	6.0%	0.38 [-0.35, 1.12]
Antonino 2001	15.67	1.83	30	13.58	1.81	30	0.0%	1.13 [0.59, 1.68]
Aris 2012	15.81	4.59	40	16.54	4.69	44	17.7%	-0.16 [-0.58, 0.27]
B.G 2001	20.18	7.73	31	17.87	5.7	30	12.8%	0.33 [-0.17, 0.84]
Claire 2007	18.7	8.7	9	15.8	7.1	9	3.8%	0.35 [-0.59, 1.28]
Enderle 2000	12.6	5.7	10	11.2	6.9	10	4.2%	0.21 [-0.67, 1.09]
Marie(2) 1998	11.3	1.2	9	12.7	1.3	8	0.0%	-1.07 [-2.10, -0.03]
Marie 1998	12.1	0.1	9	12.7	1.3	8	0.0%	-0.64 [-1.62, 0.34]
Mark K 2005	11.5	6.7	28	12.9	7.2	33	12.8%	-0.20 [-0.70, 0.31]
Paola 2011	21.3	3.13	20	15.7	3.51	20	0.0%	1.65 [0.92, 2.38]
PERT 2019	30	0.9	86	30	0.9	86	36.5%	0.00 [-0.30, 0.30]
W.Marchien 1999	14.4	1.1	14	12.4	1.8	13	0.0%	1.31 [0.47, 2.16]
**Total (95% CI)**			**234**			**240**	**100.0%**	**0.03 [-0.15, 0.22]**

Heterogeneity: Tau² = 0.00; Chi² = 4.44, df = 7 (P = 0.73); I² = 0%
Test for overall effect: Z = 0.38 (P = 0.71)

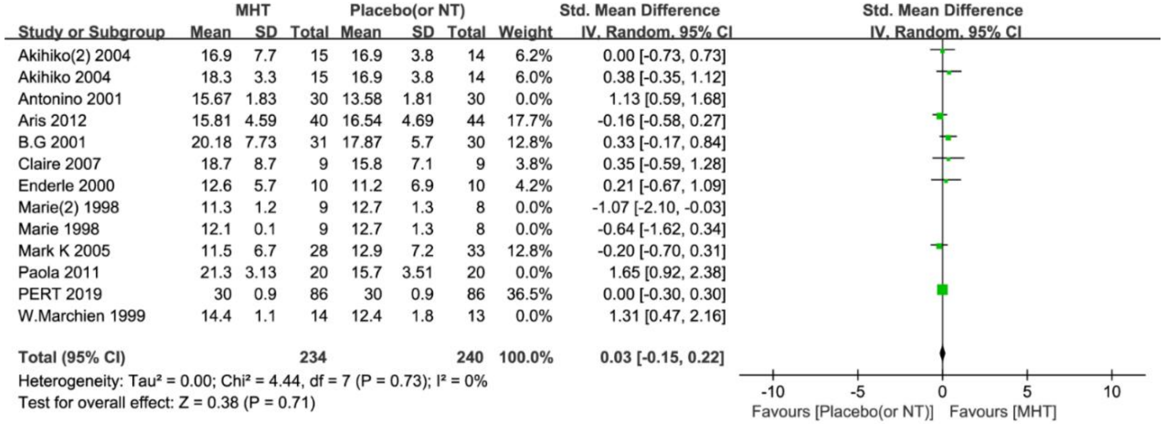

Figure S2A: FMD sensitivity analysis; Figure S2B: NMD sensitivity analysis

Figure S3 Funnel plots

(A)

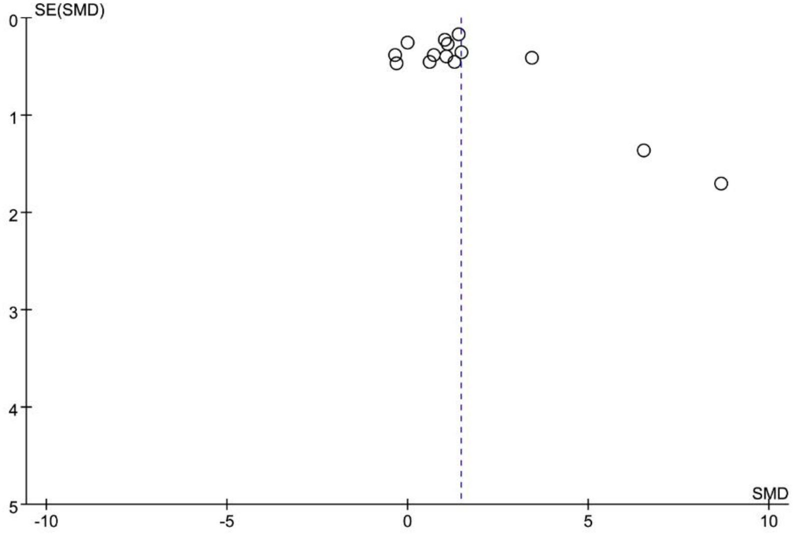

(B)

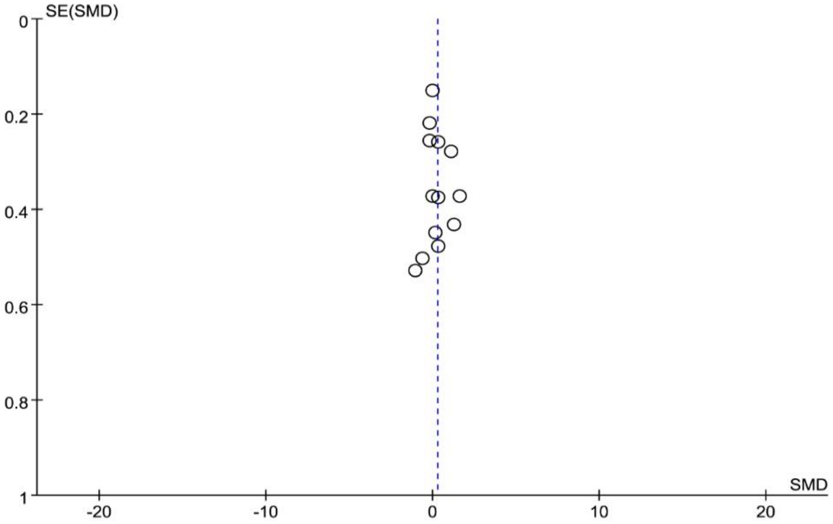

Figure S3A: FMD funnel plot; Figure S3B: NMD funnel plot

Figure S4 Subgroup analysis of different MHT treatment durations

(A)

Study or Subgroup	MHT Mean	MHT SD	MHT Total	Placebo(or NT) Mean	Placebo(or NT) SD	Placebo(or NT) Total	Weight	Std. Mean Difference IV, Random, 95% CI
**27.1.1 Duration of treatment<1 month**								
Enderle 2000	5	2.8	10	3.5	1.7	10	7.3%	0.62 [-0.28, 1.52]
J.E.B 2003	3.4	0.45	9	0.5	0.03	9	2.4%	8.66 [5.33, 11.99]
**Subtotal (95% CI)**			**19**			**19**	**9.7%**	**4.47 [-3.40, 12.35]**
Heterogeneity: Tau² = 30.77; Chi² = 20.84, df = 1 (P < 0.00001); I² = 95%								
Test for overall effect: Z = 1.11 (P = 0.27)								
**27.1.2 Duration of treatment ≥1 month and <6 month**								
Akihiko(2) 2004	7.5	3.3	15	4.9	3.6	14	7.7%	0.73 [-0.02, 1.49]
Akihiko 2004	8.9	3.7	15	4.9	3.6	14	7.6%	1.06 [0.28, 1.85]
Aris 2012	7.59	2.63	40	4.91	2.49	40	8.3%	1.04 [0.57, 1.50]
Claire 2007	4.7	4.8	9	6.2	4.7	9	7.2%	-0.30 [-1.23, 0.63]
Kerrie 2013	6.2	0.6	15	5.4	0.6	10	7.3%	1.29 [0.40, 2.18]
Marie(2) 1998	9.6	0.8	9	4.7	0.6	8	3.2%	6.52 [3.84, 9.19]
Marie 1998	11.1	0.1	9	4.7	0.6	8	1.0%	14.59 [8.91, 20.27]
**Subtotal (95% CI)**			**112**			**103**	**42.4%**	**1.64 [0.62, 2.66]**
Heterogeneity: Tau² = 1.38; Chi² = 46.80, df = 6 (P < 0.00001); I² = 87%								
Test for overall effect: Z = 3.15 (P = 0.002)								
**27.1.3 Duration of treatment≥6 month and <12month**								
Antonino 2001	11.55	1.46	30	7.17	1.03	30	7.5%	3.42 [2.61, 4.23]
B.G 2001	10.58	3.94	31	6.26	3.69	30	8.2%	1.12 [0.57, 1.66]
Paola 2011	12.75	2.57	20	9.09	2.25	20	7.8%	1.49 [0.78, 2.19]
**Subtotal (95% CI)**			**81**			**80**	**23.5%**	**1.98 [0.68, 3.29]**
Heterogeneity: Tau² = 1.21; Chi² = 22.10, df = 2 (P < 0.0001); I² = 91%								
Test for overall effect: Z = 2.97 (P = 0.003)								
**27.1.4 Duration of treatment≥12 month**								
Mark K 2005	4.1	6.5	28	4.1	5.2	33	8.2%	0.00 [-0.50, 0.50]
PERT 2019	7.1	0.5	86	6.4	0.5	86	8.5%	1.39 [1.06, 1.73]
W.Marchien 1999	6.7	0.9	14	7.1	1.3	13	7.7%	-0.35 [-1.11, 0.41]
**Subtotal (95% CI)**			**128**			**132**	**24.4%**	**0.38 [-0.77, 1.53]**
Heterogeneity: Tau² = 0.94; Chi² = 30.58, df = 2 (P < 0.00001); I² = 93%								
Test for overall effect: Z = 0.65 (P = 0.52)								
**Total (95% CI)**			**340**			**334**	**100.0%**	**1.46 [0.86, 2.07]**
Heterogeneity: Tau² = 1.10; Chi² = 135.95, df = 14 (P < 0.00001); I² = 90%								
Test for overall effect: Z = 4.73 (P < 0.00001)								
Test for subgroup differences: Chi² = 4.57, df = 3 (P = 0.21), I² = 34.4%								

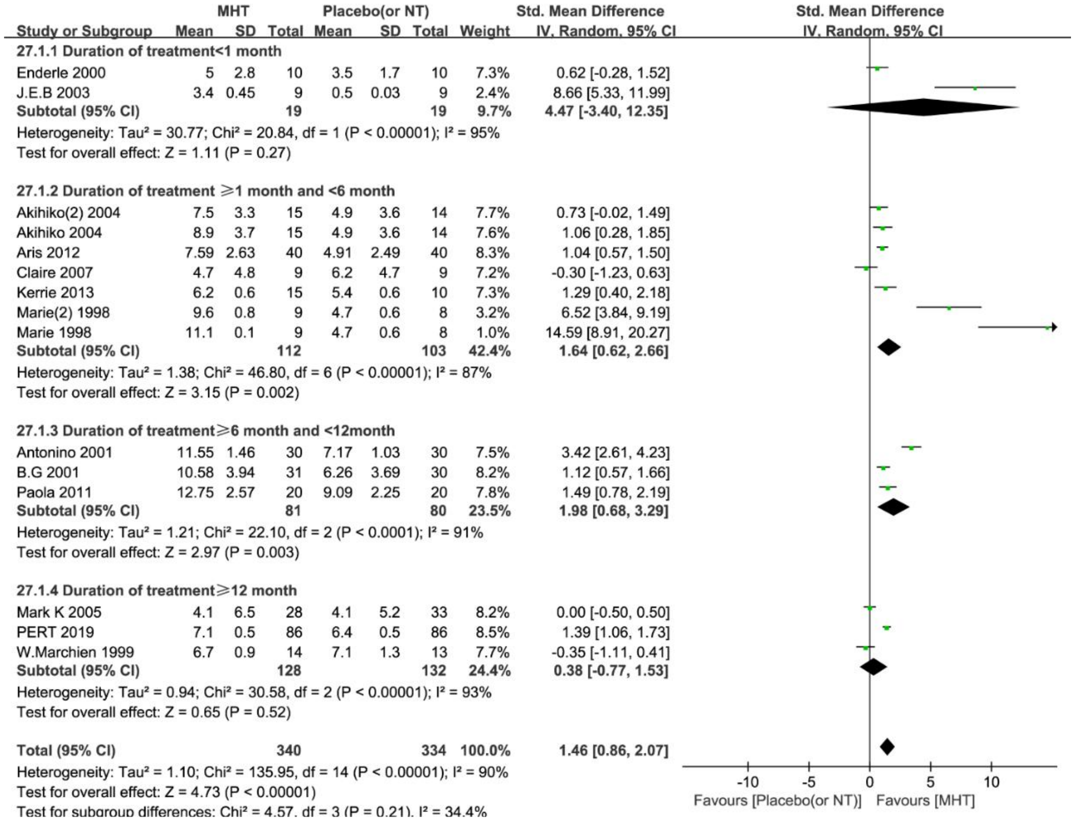

(B)

Study or Subgroup	MHT Mean	MHT SD	MHT Total	Placebo(or NT) Mean	Placebo(or NT) SD	Placebo(or NT) Total	Weight	Std. Mean Difference IV, Random, 95% CI
**28.1.1 Duration of treatment<1month**								
Enderle 2000	12.6	5.7	10	11.2	6.9	10	6.5%	0.21 [-0.67, 1.09]
**Subtotal (95% CI)**			**10**			**10**	**6.5%**	**0.21 [-0.67, 1.09]**
Heterogeneity: Not applicable								
Test for overall effect: Z = 0.47 (P = 0.64)								
**28.1.2 Duration of treatment≥1 month and <6 month**								
Akihiko(2) 2004	16.9	7.7	15	16.9	3.8	14	7.5%	0.00 [-0.73, 0.73]
Akihiko 2004	18.3	3.3	15	16.9	3.8	14	7.4%	0.38 [-0.35, 1.12]
Aris 2012	15.81	4.59	40	16.54	4.69	44	9.5%	-0.16 [-0.58, 0.27]
Claire 2007	18.7	8.7	9	15.8	7.1	9	6.2%	0.35 [-0.59, 1.28]
Marie(2) 1998	11.3	1.2	9	12.7	1.3	8	5.6%	-1.07 [-2.10, -0.03]
Marie 1998	12.1	0.1	9	12.7	1.3	8	5.9%	-0.64 [-1.62, 0.34]
**Subtotal (95% CI)**			**97**			**97**	**42.2%**	**-0.12 [-0.49, 0.24]**
Heterogeneity: Tau² = 0.06; Chi² = 7.16, df = 5 (P = 0.21); I² = 30%								
Test for overall effect: Z = 0.65 (P = 0.52)								
**28.1.3 Duration of treatment≥6 month and <12 month**								
Antonino 2001	15.67	1.83	30	13.58	1.81	30	8.7%	1.13 [0.59, 1.68]
B.G 2001	20.18	7.73	31	17.87	5.7	30	9.0%	0.33 [-0.17, 0.84]
Paola 2011	21.3	3.13	20	15.7	3.51	20	7.5%	1.65 [0.92, 2.38]
**Subtotal (95% CI)**			**81**			**80**	**25.2%**	**1.01 [0.26, 1.75]**
Heterogeneity: Tau² = 0.34; Chi² = 9.58, df = 2 (P = 0.008); I² = 79%								
Test for overall effect: Z = 2.66 (P = 0.008)								
**28.1.4 Duration of treatment≥12 month**								
Mark K 2005	11.5	6.7	28	12.9	7.2	33	9.0%	-0.20 [-0.70, 0.31]
PERT 2019	30	0.9	86	30	0.9	86	10.3%	0.00 [-0.30, 0.30]
W.Marchien 1999	14.4	1.1	14	12.4	1.8	13	6.7%	1.31 [0.47, 2.16]
**Subtotal (95% CI)**			**128**			**132**	**26.0%**	**0.26 [-0.39, 0.91]**
Heterogeneity: Tau² = 0.25; Chi² = 9.60, df = 2 (P = 0.008); I² = 79%								
Test for overall effect: Z = 0.79 (P = 0.43)								
**Total (95% CI)**			**316**			**319**	**100.0%**	**0.27 [-0.08, 0.62]**
Heterogeneity: Tau² = 0.29; Chi² = 49.25, df = 12 (P < 0.00001); I² = 76%								
Test for overall effect: Z = 1.54 (P = 0.12)								
Test for subgroup differences: Chi² = 7.36, df = 3 (P = 0.06), I² = 59.2%								

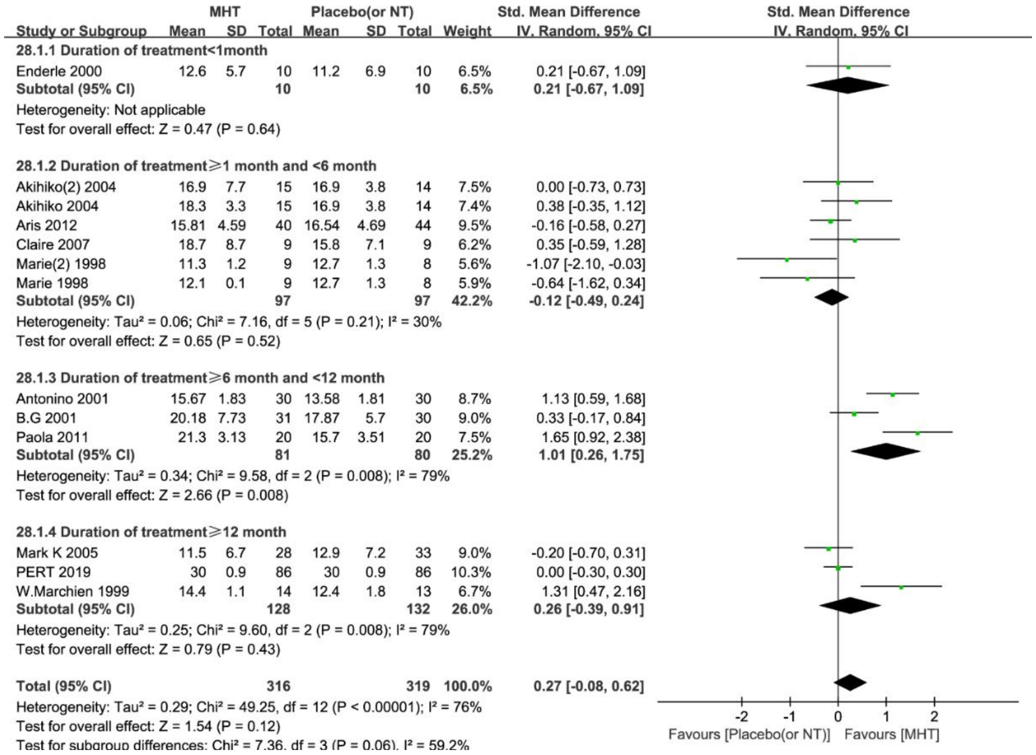

Figure S4A: Effect of different MHT treatment durations on FMD; Figure S4B: Effect of different MHT treatment durations on NMD

Figure S5 Subgroup analysis of MHT onset time

(A)

Study or Subgroup	MHT Events	MHT Total	Placebo(or NT) Events	Placebo(or NT) Total	Weight	Risk Ratio M-H, Fixed, 95% CI
**9.1.1 onset time of MHT therapy after menopause(<10 years)**						
DOPS 2012	15	502	26	504	3.7%	0.58 [0.31, 1.08]
ERT II 1979	3	84	7	84	1.0%	0.43 [0.11, 1.60]
KEEPS(2) 2014	2	230	1	275	0.1%	2.39 [0.22, 26.20]
KEEPS 2014	1	222	1	275	0.1%	1.24 [0.08, 19.69]
Piret 2006	1	404	1	373	0.1%	0.92 [0.06, 14.71]
WHI 2002	14	826	21	817	3.0%	0.66 [0.34, 1.29]
WHI II 2004	39	2782	46	2712	6.6%	0.83 [0.54, 1.26]
**Subtotal (95% CI)**		**5050**		**5040**	**14.6%**	**0.72 [0.54, 0.97]**
Total events	75		103			
Heterogeneity: Chi² = 2.68, df = 6 (P = 0.85); I² = 0%						
Test for overall effect: Z = 2.17 (P = 0.03)						
**9.1.2 onset time of MHT therapy after menopause(>10 years)**						
Catherine 2001	48	337	41	327	5.9%	1.14 [0.77, 1.67]
David M(2) 2000	3	104	6	105	0.8%	0.50 [0.13, 1.97]
David M 2000	8	100	6	105	0.8%	1.40 [0.50, 3.89]
ESPRIT 2002	21	513	30	504	4.3%	0.69 [0.40, 1.18]
Greenspan 2005	1	187	2	186	0.3%	0.50 [0.05, 5.44]
HERS 1998	131	1380	123	1383	17.3%	1.07 [0.84, 1.35]
STOP IT 2001	1	123	1	121	0.1%	0.98 [0.06, 15.55]
WAVE 2002	14	210	8	213	1.1%	1.77 [0.76, 4.14]
WHI 2002	177	4897	167	4797	23.8%	1.04 [0.84, 1.28]
WHI II 2004	232	3667	222	3819	30.7%	1.09 [0.91, 1.30]
WHISP 2006	1	49	2	51	0.3%	0.52 [0.05, 5.56]
**Subtotal (95% CI)**		**11567**		**11611**	**85.4%**	**1.06 [0.95, 1.18]**
Total events	637		608			
Heterogeneity: Chi² = 6.26, df = 10 (P = 0.79); I² = 0%						
Test for overall effect: Z = 0.99 (P = 0.32)						
**Total (95% CI)**		**16617**		**16651**	**100.0%**	**1.01 [0.91, 1.11]**
Total events	712		711			
Heterogeneity: Chi² = 14.59, df = 17 (P = 0.63); I² = 0%						
Test for overall effect: Z = 0.13 (P = 0.89)						
Test for subgroup differences: Chi² = 5.67, df = 1 (P = 0.02), I² = 82.4%						

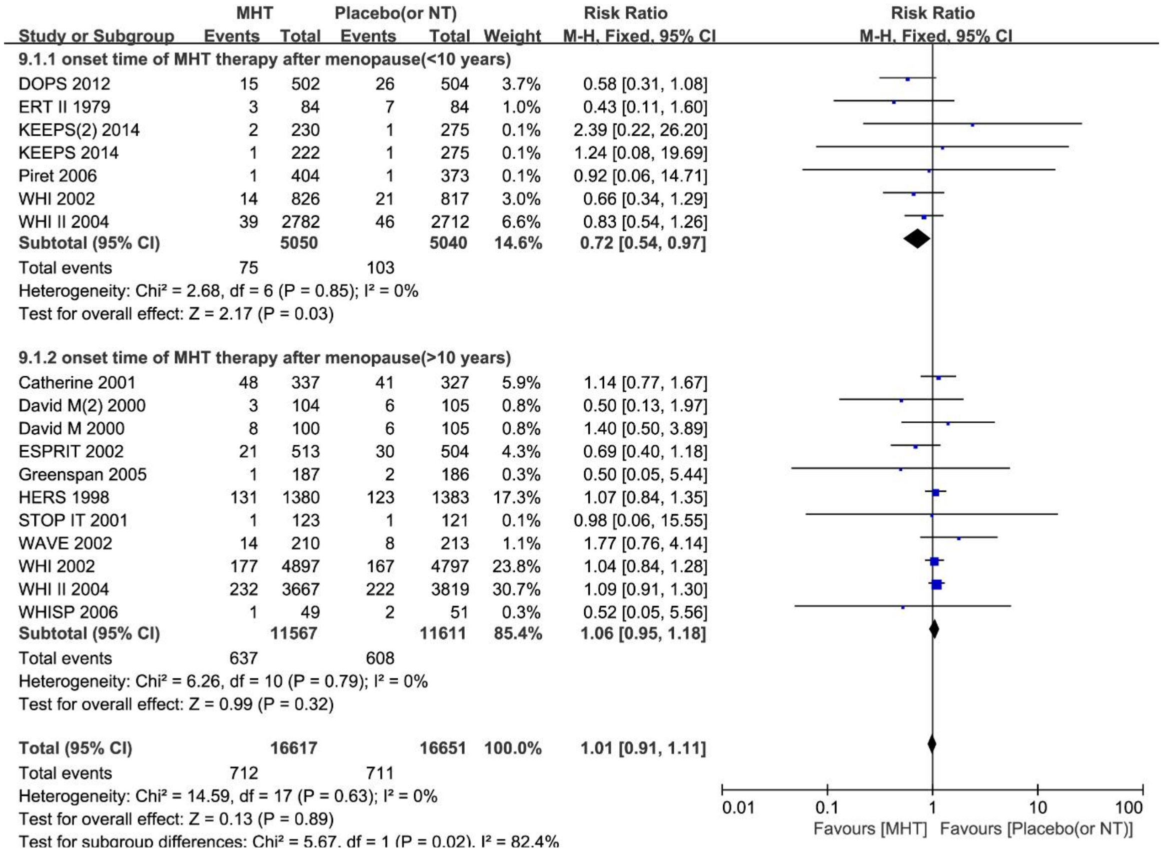

(B)

Study or Subgroup	MHT Events	MHT Total	Placebo(or NT) Events	Placebo(or NT) Total	Weight	Risk Ratio M-H, Fixed, 95% CI
**12.1.1 onset time of MHT therapy after menopause(<10 years)**						
DOPS 2012	6	502	22	504	3.8%	0.27 [0.11, 0.67]
ERT II 1979	1	84	3	84	0.5%	0.33 [0.04, 3.14]
WHI 2002	31	2782	35	2712	6.2%	0.86 [0.53, 1.40]
WHI II 2004	8	826	16	817	2.8%	0.49 [0.21, 1.15]
**Subtotal (95% CI)**		**4194**		**4117**	**13.4%**	**0.60 [0.41, 0.86]**
Total events	46		76			
Heterogeneity: Chi² = 5.64, df = 3 (P = 0.13); I² = 47%						
Test for overall effect: Z = 2.79 (P = 0.005)						
**12.1.2 onset time of MHT therapy after menopause(≥10 years)**						
Catherine 2001	25	337	25	327	4.4%	0.97 [0.57, 1.65]
HERS 1998	192	1380	193	1383	33.8%	1.00 [0.83, 1.20]
WAVE 2002	12	210	10	213	1.7%	1.22 [0.54, 2.76]
WHI 2002	143	4897	100	4797	17.7%	1.40 [1.09, 1.80]
WHI II 2004	164	3667	161	3819	27.6%	1.06 [0.86, 1.31]
WHISP 2006	3	49	8	51	1.4%	0.39 [0.11, 1.39]
**Subtotal (95% CI)**		**10540**		**10590**	**86.6%**	**1.09 [0.97, 1.23]**
Total events	539		497			
Heterogeneity: Chi² = 7.53, df = 5 (P = 0.18); I² = 34%						
Test for overall effect: Z = 1.49 (P = 0.14)						
**Total (95% CI)**		**14734**		**14707**	**100.0%**	**1.03 [0.92, 1.15]**
Total events	585		573			
Heterogeneity: Chi² = 21.20, df = 9 (P = 0.01); I² = 58%						
Test for overall effect: Z = 0.46 (P = 0.64)						
Test for subgroup differences: Chi² = 9.68, df = 1 (P = 0.002), I² = 89.7%						

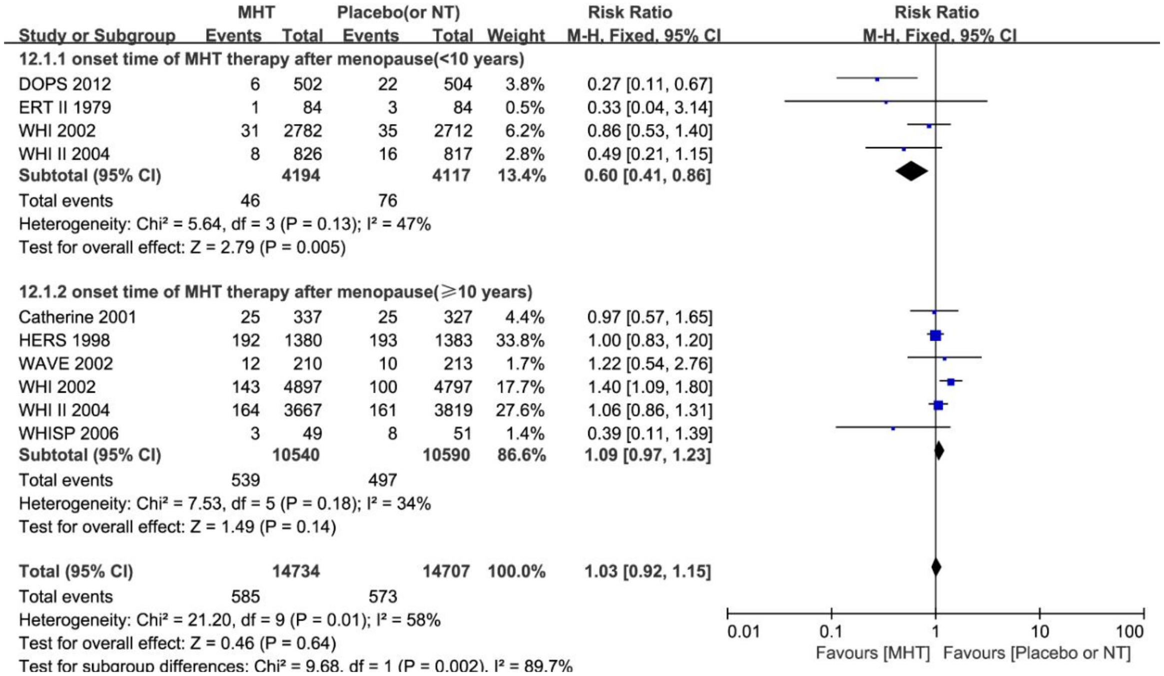

Figure S5A: Effect of MHT onset time on all-cause death; Figure S5B: Effect of MHT onset time on cardiovascular events

(C)

Study or Subgroup	MHT Events	MHT Total	Placebo or NT Events	Placebo or NT Total	Weight	Risk Ratio M-H, Fixed, 95% CI
**15.1.1 onset time of MHT therapy after menopause(<10 years)**						
DOPS 2012	11	502	14	504	3.5%	0.79 [0.36, 1.72]
KEEPS(2) 2014	1	222	1	275	0.2%	1.24 [0.08, 19.69]
KEEPS 2014	1	230	1	275	0.2%	1.20 [0.08, 19.01]
Piret 2006	1	404	1	373	0.3%	0.92 [0.06, 14.71]
WHI 2002	24	2782	15	2712	3.8%	1.56 [0.82, 2.97]
WHI II 2004	17	826	8	817	2.0%	2.10 [0.91, 4.84]
**Subtotal (95% CI)**		**4966**		**4956**	**9.9%**	**1.37 [0.91, 2.05]**
Total events	55		40			
Heterogeneity: Chi² = 3.18, df = 5 (P = 0.67); I² = 0%						
Test for overall effect: Z = 1.52 (P = 0.13)						
**15.1.2 onset time of MHT therapy after menopause(⩾10 years)**						
Catherine 2001	63	337	56	327	14.1%	1.09 [0.79, 1.51]
David M(2) 2000	6	104	6	105	1.5%	1.01 [0.34, 3.03]
David M 2000	5	100	6	105	1.5%	0.88 [0.28, 2.78]
HERS 1998	108	1380	96	1383	23.8%	1.13 [0.87, 1.47]
STOP IT 2001	4	121	3	123	0.7%	1.36 [0.31, 5.93]
WAVE 2002	4	103	3	108	0.7%	1.40 [0.32, 6.10]
WHI 2002	113	4897	90	4797	22.5%	1.23 [0.94, 1.62]
WHI II 2004	129	3669	102	3819	24.8%	1.32 [1.02, 1.70]
WHISP 2006	1	49	2	51	0.5%	0.52 [0.05, 5.56]
**Subtotal (95% CI)**		**10760**		**10818**	**90.1%**	**1.19 [1.04, 1.37]**
Total events	433		364			
Heterogeneity: Chi² = 1.99, df = 8 (P = 0.98); I² = 0%						
Test for overall effect: Z = 2.58 (P = 0.010)						
**Total (95% CI)**		**15726**		**15774**	**100.0%**	**1.21 [1.07, 1.38]**
Total events	488		404			
Heterogeneity: Chi² = 5.52, df = 14 (P = 0.98); I² = 0%						
Test for overall effect: Z = 2.94 (P = 0.003)						
Test for subgroup differences: Chi² = 0.39, df = 1 (P = 0.53), I² = 0%						

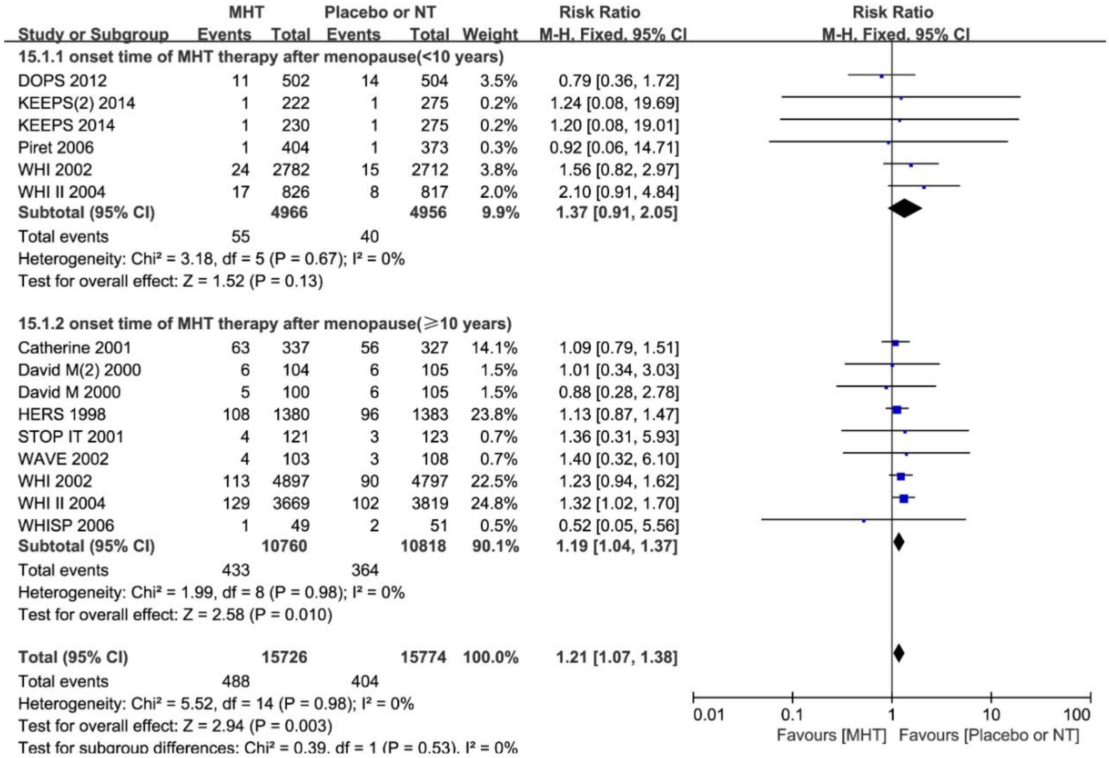

(D)

Study or Subgroup	MHT Events	MHT Total	Placebo(or NT) Events	Placebo(or NT) Total	Weight	Risk Ratio M-H, Fixed, 95% CI
**18.1.1 onset time of MHT therapy after menopause(<10 years)**						
DOPS 2012	2	502	2	504	1.0%	1.00 [0.14, 7.10]
EVTET 2000	8	71	1	69	0.5%	7.77 [1.00, 60.53]
KEEPS(2) 2014	2	222	2	275	0.9%	1.24 [0.18, 8.72]
KEEPS 2014	1	230	2	275	0.9%	0.60 [0.05, 6.55]
WHI 2002	32	2839	13	2683	6.9%	2.33 [1.22, 4.42]
WHI II 2004	20	1637	15	1673	7.6%	1.36 [0.70, 2.65]
**Subtotal (95% CI)**		**5501**		**5479**	**17.9%**	**1.85 [1.23, 2.79]**
Total events	65		35			
Heterogeneity: Chi² = 4.57, df = 5 (P = 0.47); I² = 0%						
Test for overall effect: Z = 2.94 (P = 0.003)						
**18.1.2 onset time of MHT therapy after menopause(⩾10 years)**						
Catherine 2001	3	337	4	327	2.1%	0.73 [0.16, 3.23]
David M(2) 2000	2	104	1	105	0.5%	2.02 [0.19, 21.93]
David M 2000	5	100	1	105	0.5%	5.25 [0.62, 44.16]
Greenspan 2005	2	187	1	186	0.5%	1.99 [0.18, 21.75]
HERS 1998	34	1380	12	1383	6.2%	2.84 [1.48, 5.46]
STOP IT 2001	2	121	1	123	0.5%	2.03 [0.19, 22.13]
WAVE 2002	4	210	4	213	2.0%	1.01 [0.26, 4.00]
WHI 2002	136	5667	63	5419	33.1%	2.06 [1.53, 2.78]
WHI II 2004	91	3673	71	3756	36.1%	1.31 [0.96, 1.78]
WHISP 2006	1	49	1	51	0.5%	1.04 [0.07, 16.18]
**Subtotal (95% CI)**		**11828**		**11668**	**82.1%**	**1.74 [1.44, 2.11]**
Total events	280		159			
Heterogeneity: Chi² = 9.83, df = 9 (P = 0.36); I² = 8%						
Test for overall effect: Z = 5.64 (P < 0.00001)						
**Total (95% CI)**		**17329**		**17147**	**100.0%**	**1.76 [1.48, 2.10]**
Total events	345		194			
Heterogeneity: Chi² = 14.40, df = 15 (P = 0.50); I² = 0%						
Test for overall effect: Z = 6.35 (P < 0.00001)						
Test for subgroup differences: Chi² = 0.07, df = 1 (P = 0.79), I² = 0%						

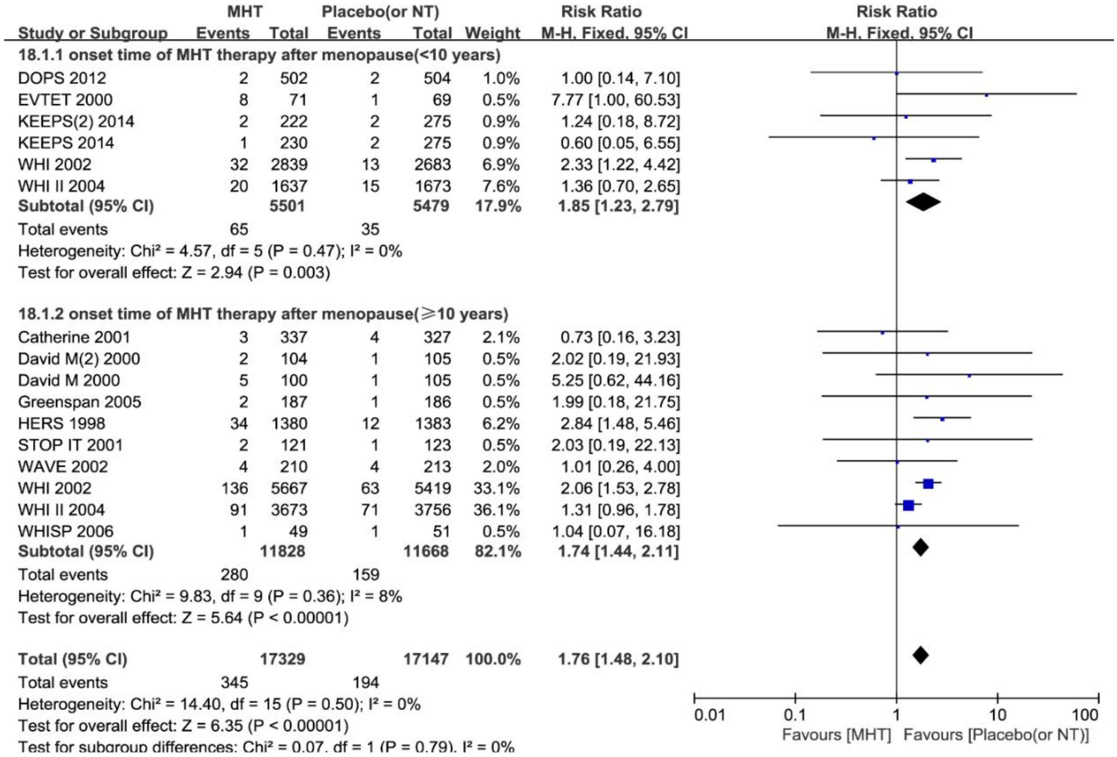

Figure S5C: Effect of MHT onset time on stroke; Figure S5D: Effect of MHT onset time on venous thromboembolism

(E)

Study or Subgroup	MHT Mean	MHT SD	MHT Total	Placebo(or NT) Mean	Placebo(or NT) SD	Placebo(or NT) Total	Weight	Std. Mean Difference IV, Random, 95% CI
**22.1.1 onset time of HRT therapy after menopause(<10 years)**								
Akihiko(2) 2004	7.5	3.3	15	4.9	3.6	14	6.7%	0.73 [-0.02, 1.49]
Akihiko 2004	8.9	3.7	15	4.9	3.6	14	6.6%	1.06 [0.28, 1.85]
Andrew(2) 2007	5.81	4.85	7	4.81	5.97	6	5.6%	0.17 [-0.92, 1.27]
Andrew 2007	7.69	4.79	6	4.81	5.97	6	5.4%	0.49 [-0.67, 1.65]
Antonino 2001	11.55	1.46	30	7.17	1.03	30	6.5%	3.42 [2.61, 4.23]
Aris 2012	7.59	2.63	40	4.91	2.49	40	7.6%	1.04 [0.57, 1.50]
B.G 2001	10.58	3.94	31	6.26	3.69	30	7.4%	1.12 [0.57, 1.66]
Paola 2011	12.75	2.57	20	9.09	2.25	20	6.9%	1.49 [0.78, 2.19]
PERT 2019	7.1	0.5	86	6.4	0.5	86	7.9%	1.39 [1.06, 1.73]
W.Marchien 1999	6.7	0.9	14	7.1	1.3	13	6.7%	-0.35 [-1.11, 0.41]
**Subtotal (95% CI)**			**264**			**259**	**67.1%**	**1.09 [0.58, 1.59]**
Heterogeneity: Tau² = 0.52; Chi² = 53.61, df = 9 (P < 0.00001); I² = 83%								
Test for overall effect: Z = 4.22 (P < 0.0001)								
**22.1.2 onset time of HRT therapy after menopause(≥10 years)**								
Andrew(2) 2007	3.47	3.01	12	3.8	3.6	12	6.6%	-0.10 [-0.90, 0.70]
Andrew 2007	2.74	3.8	12	3.8	3.6	12	6.5%	-0.28 [-1.08, 0.53]
Claire 2007	4.7	4.8	9	6.2	4.7	9	6.1%	-0.30 [-1.23, 0.63]
Enderle 2000	5	2.8	10	3.5	1.7	10	6.2%	0.62 [-0.28, 1.52]
Mark K 2005	4.1	6.5	28	4.1	5.2	33	7.5%	0.00 [-0.50, 0.50]
**Subtotal (95% CI)**			**71**			**76**	**32.9%**	**-0.02 [-0.34, 0.31]**
Heterogeneity: Tau² = 0.00; Chi² = 2.71, df = 4 (P = 0.61); I² = 0%								
Test for overall effect: Z = 0.10 (P = 0.92)								
**Total (95% CI)**			**335**			**335**	**100.0%**	**0.72 [0.27, 1.18]**
Heterogeneity: Tau² = 0.65; Chi² = 95.10, df = 14 (P < 0.00001); I² = 85%								
Test for overall effect: Z = 3.12 (P = 0.002)								
Test for subgroup differences: Chi² = 12.99, df = 1 (P = 0.0003), I² = 92.3%								

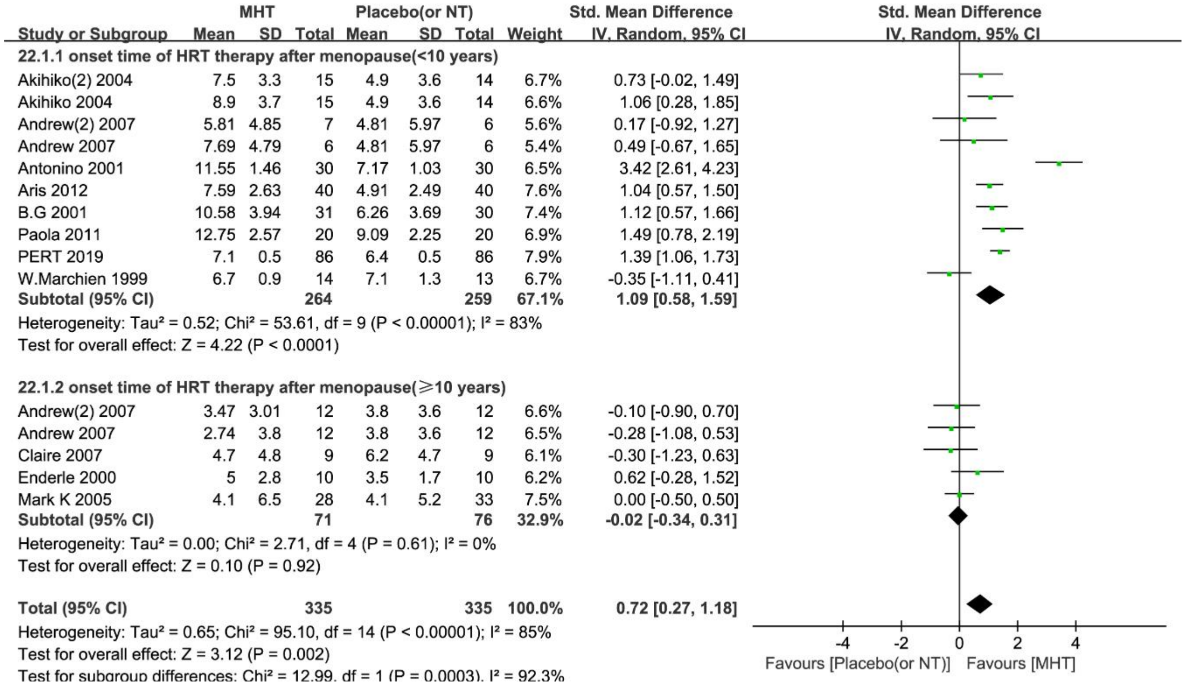

(F)

Study or Subgroup	MHT Mean	MHT SD	MHT Total	Placebo(or NT) Mean	Placebo(or NT) SD	Placebo(or NT) Total	Weight	Std. Mean Difference IV, Random, 95% CI
**25.1.1 onset time of MHT therapy after menopause(<10 years)**								
Akihiko(2) 2004	16.9	7.7	15	16.9	3.8	14	6.5%	0.00 [-0.73, 0.73]
Akihiko 2004	18.3	3.3	15	16.9	3.8	14	6.5%	0.38 [-0.35, 1.12]
Andrew(2) 2007	21.84	6.54	7	20.25	8.91	6	4.3%	0.19 [-0.90, 1.29]
Andrew 2007	20.85	9.57	6	20.25	8.91	6	4.1%	0.06 [-1.07, 1.19]
Antonino 2001	15.67	1.83	30	13.58	1.81	30	8.0%	1.13 [0.59, 1.68]
Aris 2012	15.81	4.59	40	16.54	4.69	44	9.0%	-0.16 [-0.58, 0.27]
B.G 2001	20.18	7.73	31	17.87	5.7	30	8.4%	0.33 [-0.17, 0.84]
Paola 2011	21.3	3.13	20	15.7	3.51	20	6.5%	1.65 [0.92, 2.38]
PERT 2019	30	0.9	86	30	0.9	86	10.1%	0.00 [-0.30, 0.30]
W.Marchien 1999	14.4	1.1	14	12.4	1.8	13	5.7%	1.31 [0.47, 2.16]
**Subtotal (95% CI)**			**264**			**263**	**69.0%**	**0.48 [0.09, 0.87]**
Heterogeneity: Tau² = 0.27; Chi² = 36.77, df = 9 (P < 0.0001); I² = 76%								
Test for overall effect: Z = 2.39 (P = 0.02)								
**25.1.2 onset time of MHT therapy after menopause(≥10 years)**								
Andrew(2) 2007	14.69	7.2	12	13.35	9.35	12	6.0%	0.16 [-0.65, 0.96]
Andrew 2007	13.71	8.3	12	13.35	9.35	12	6.0%	0.04 [-0.76, 0.84]
Claire 2007	18.7	8.7	9	15.8	7.1	9	5.1%	0.35 [-0.59, 1.28]
Enderle 2000	12.6	5.7	10	11.2	6.9	10	5.5%	0.21 [-0.67, 1.09]
Mark K 2005	11.5	6.7	28	12.9	7.2	33	8.4%	-0.20 [-0.70, 0.31]
**Subtotal (95% CI)**			**71**			**76**	**31.0%**	**0.02 [-0.30, 0.35]**
Heterogeneity: Tau² = 0.00; Chi² = 1.49, df = 4 (P = 0.83); I² = 0%								
Test for overall effect: Z = 0.13 (P = 0.90)								
**Total (95% CI)**			**335**			**339**	**100.0%**	**0.35 [0.06, 0.63]**
Heterogeneity: Tau² = 0.19; Chi² = 40.63, df = 14 (P = 0.0002); I² = 66%								
Test for overall effect: Z = 2.38 (P = 0.02)								
Test for subgroup differences: Chi² = 3.08, df = 1 (P = 0.08), I² = 67.6%								

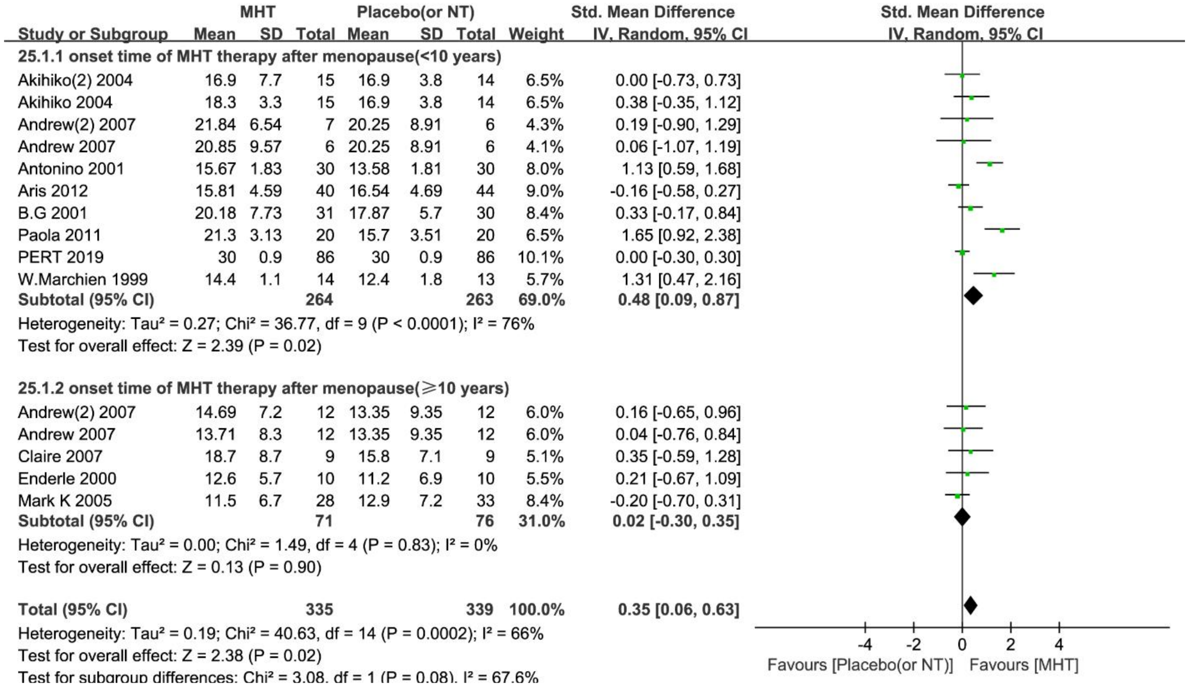

Figure S5E: Effect of MHT onset time on FMD; Figure S5F: Effect of MHT onset time on NMD

Figure S6 Subgroup analysis of primary prevention and secondary prevention of MHT

(A)

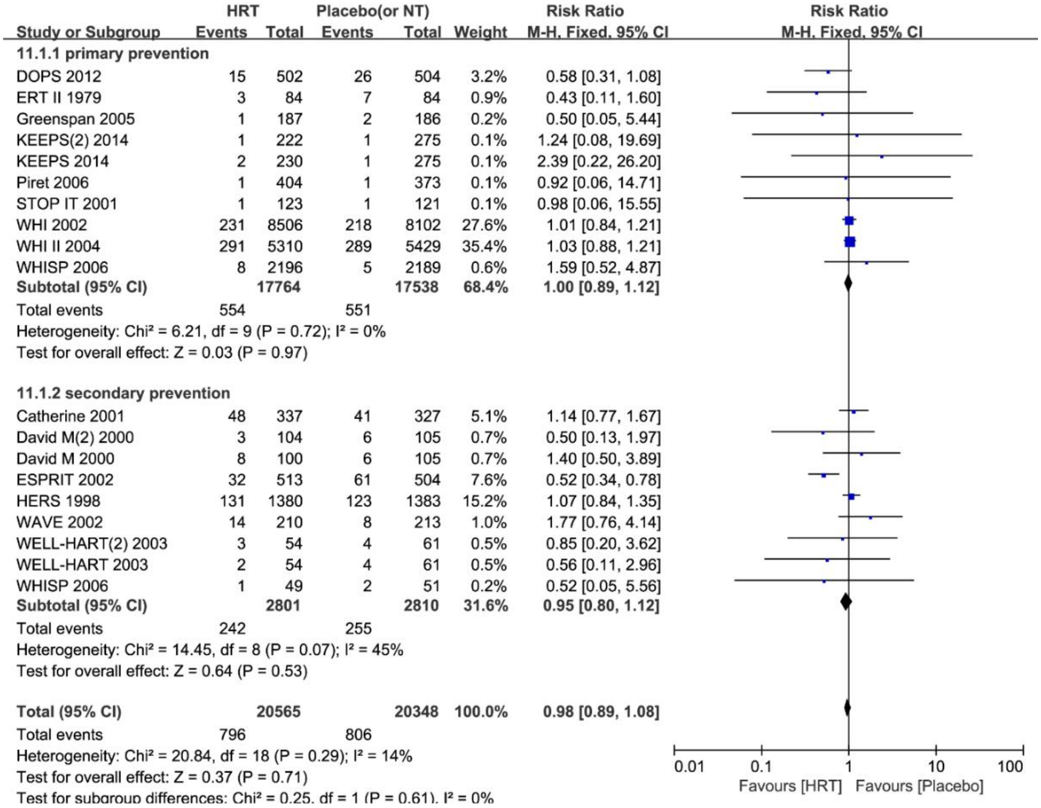

Study or Subgroup	HRT Events	HRT Total	Placebo(or NT) Events	Placebo(or NT) Total	Weight	Risk Ratio M-H, Fixed, 95% CI
**11.1.1 primary prevention**						
DOPS 2012	15	502	26	504	3.2%	0.58 [0.31, 1.08]
ERT II 1979	3	84	7	84	0.9%	0.43 [0.11, 1.60]
Greenspan 2005	1	187	2	186	0.2%	0.50 [0.05, 5.44]
KEEPS(2) 2014	1	222	1	275	0.1%	1.24 [0.08, 19.69]
KEEPS 2014	2	230	1	275	0.1%	2.39 [0.22, 26.20]
Piret 2006	1	404	1	373	0.1%	0.92 [0.06, 14.71]
STOP IT 2001	1	123	1	121	0.1%	0.98 [0.06, 15.55]
WHI 2002	231	8506	218	8102	27.6%	1.01 [0.84, 1.21]
WHI II 2004	291	5310	289	5429	35.4%	1.03 [0.88, 1.21]
WHISP 2006	8	2196	5	2189	0.6%	1.59 [0.52, 4.87]
**Subtotal (95% CI)**		**17764**		**17538**	**68.4%**	**1.00 [0.89, 1.12]**
Total events	554		551			
Heterogeneity: Chi² = 6.21, df = 9 (P = 0.72); I² = 0%						
Test for overall effect: Z = 0.03 (P = 0.97)						
**11.1.2 secondary prevention**						
Catherine 2001	48	337	41	327	5.1%	1.14 [0.77, 1.67]
David M(2) 2000	3	104	6	105	0.7%	0.50 [0.13, 1.97]
David M 2000	8	100	6	105	0.7%	1.40 [0.50, 3.89]
ESPRIT 2002	32	513	61	504	7.6%	0.52 [0.34, 0.78]
HERS 1998	131	1380	123	1383	15.2%	1.07 [0.84, 1.35]
WAVE 2002	14	210	8	213	1.0%	1.77 [0.76, 4.14]
WELL-HART(2) 2003	3	54	4	61	0.5%	0.85 [0.20, 3.62]
WELL-HART 2003	2	54	4	61	0.5%	0.56 [0.11, 2.96]
WHISP 2006	1	49	2	51	0.2%	0.52 [0.05, 5.56]
**Subtotal (95% CI)**		**2801**		**2810**	**31.6%**	**0.95 [0.80, 1.12]**
Total events	242		255			
Heterogeneity: Chi² = 14.45, df = 8 (P = 0.07); I² = 45%						
Test for overall effect: Z = 0.64 (P = 0.53)						
**Total (95% CI)**		**20565**		**20348**	**100.0%**	**0.98 [0.89, 1.08]**
Total events	796		806			
Heterogeneity: Chi² = 20.84, df = 18 (P = 0.29); I² = 14%						
Test for overall effect: Z = 0.37 (P = 0.71)						
Test for subgroup differences: Chi² = 0.25, df = 1 (P = 0.61), I² = 0%						

(B)

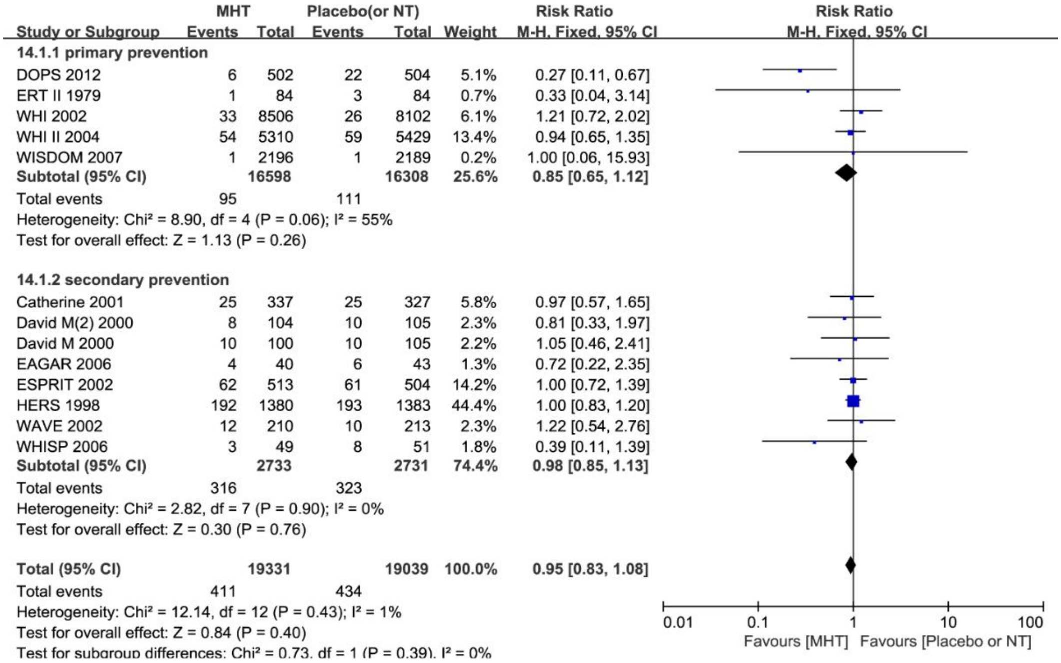

Study or Subgroup	MHT Events	MHT Total	Placebo(or NT) Events	Placebo(or NT) Total	Weight	Risk Ratio M-H, Fixed, 95% CI
**14.1.1 primary prevention**						
DOPS 2012	6	502	22	504	5.1%	0.27 [0.11, 0.67]
ERT II 1979	1	84	3	84	0.7%	0.33 [0.04, 3.14]
WHI 2002	33	8506	26	8102	6.1%	1.21 [0.72, 2.02]
WHI II 2004	54	5310	59	5429	13.4%	0.94 [0.65, 1.35]
WISDOM 2007	1	2196	1	2189	0.2%	1.00 [0.06, 15.93]
**Subtotal (95% CI)**		**16598**		**16308**	**25.6%**	**0.85 [0.65, 1.12]**
Total events	95		111			
Heterogeneity: Chi² = 8.90, df = 4 (P = 0.06); I² = 55%						
Test for overall effect: Z = 1.13 (P = 0.26)						
**14.1.2 secondary prevention**						
Catherine 2001	25	337	25	327	5.8%	0.97 [0.57, 1.65]
David M(2) 2000	8	104	10	105	2.3%	0.81 [0.33, 1.97]
David M 2000	10	100	10	105	2.2%	1.05 [0.46, 2.41]
EAGAR 2006	4	40	6	43	1.3%	0.72 [0.22, 2.35]
ESPRIT 2002	62	513	61	504	14.2%	1.00 [0.72, 1.39]
HERS 1998	192	1380	193	1383	44.4%	1.00 [0.83, 1.20]
WAVE 2002	12	210	10	213	2.3%	1.22 [0.54, 2.76]
WHISP 2006	3	49	8	51	1.8%	0.39 [0.11, 1.39]
**Subtotal (95% CI)**		**2733**		**2731**	**74.4%**	**0.98 [0.85, 1.13]**
Total events	316		323			
Heterogeneity: Chi² = 2.82, df = 7 (P = 0.90); I² = 0%						
Test for overall effect: Z = 0.30 (P = 0.76)						
**Total (95% CI)**		**19331**		**19039**	**100.0%**	**0.95 [0.83, 1.08]**
Total events	411		434			
Heterogeneity: Chi² = 12.14, df = 12 (P = 0.43); I² = 1%						
Test for overall effect: Z = 0.84 (P = 0.40)						
Test for subgroup differences: Chi² = 0.73, df = 1 (P = 0.39), I² = 0%						

Figure S6A: Effect of different MHT prevention modes on all-cause death; Figure S6B: Effect of different MHT prevention modes on cardiovascular events

(C)

Study or Subgroup	MHT Events	MHT Total	Placcebo or NT Events	Placcebo or NT Total	Weight	Risk Ratio M-H, Fixed, 95% CI
**17.1.1 primary prevention**						
DOPS 2012	11	502	14	504	3.7%	0.79 [0.36, 1.72]
EPAT 2001	2	111	1	111	0.3%	2.00 [0.18, 21.74]
KEEPS(2) 2014	1	222	1	275	0.2%	1.24 [0.08, 19.69]
KEEPS 2014	1	230	1	275	0.2%	1.20 [0.08, 19.01]
Piret 2006	1	404	1	373	0.3%	0.92 [0.06, 14.71]
STOP IT 2001	4	121	3	123	0.8%	1.36 [0.31, 5.93]
WHI 2002	110	8506	72	8102	19.3%	1.46 [1.08, 1.96]
WHI II 2004	129	5310	99	5429	25.6%	1.33 [1.03, 1.73]
**Subtotal (95% CI)**		**15406**		**15192**	**50.4%**	**1.34 [1.11, 1.61]**
Total events	259		192			
Heterogeneity: Chi² = 2.26, df = 7 (P = 0.94); I² = 0%						
Test for overall effect: Z = 3.10 (P = 0.002)						
**17.1.2 secondary prevention**						
Catherine 2001	63	337	56	327	14.9%	1.09 [0.79, 1.51]
David M(2) 2000	6	104	6	105	1.6%	1.01 [0.34, 3.03]
David M 2000	5	100	6	105	1.5%	0.88 [0.28, 2.78]
ESPRIT 2002	25	513	19	504	5.0%	1.29 [0.72, 2.32]
HERS 1998	108	1380	96	1383	25.1%	1.13 [0.87, 1.47]
WAVE 2002	9	210	4	213	1.0%	2.28 [0.71, 7.30]
WHISP 2006	1	49	2	51	0.5%	0.52 [0.05, 5.56]
**Subtotal (95% CI)**		**2693**		**2688**	**49.6%**	**1.14 [0.95, 1.37]**
Total events	217		189			
Heterogeneity: Chi² = 2.29, df = 6 (P = 0.89); I² = 0%						
Test for overall effect: Z = 1.38 (P = 0.17)						
**Total (95% CI)**		**18099**		**17880**	**100.0%**	**1.24 [1.09, 1.41]**
Total events	476		381			
Heterogeneity: Chi² = 6.09, df = 14 (P = 0.96); I² = 0%						
Test for overall effect: Z = 3.23 (P = 0.001)						
Test for subgroup differences: Chi² = 1.47, df = 1 (P = 0.23), I² = 32.1%						

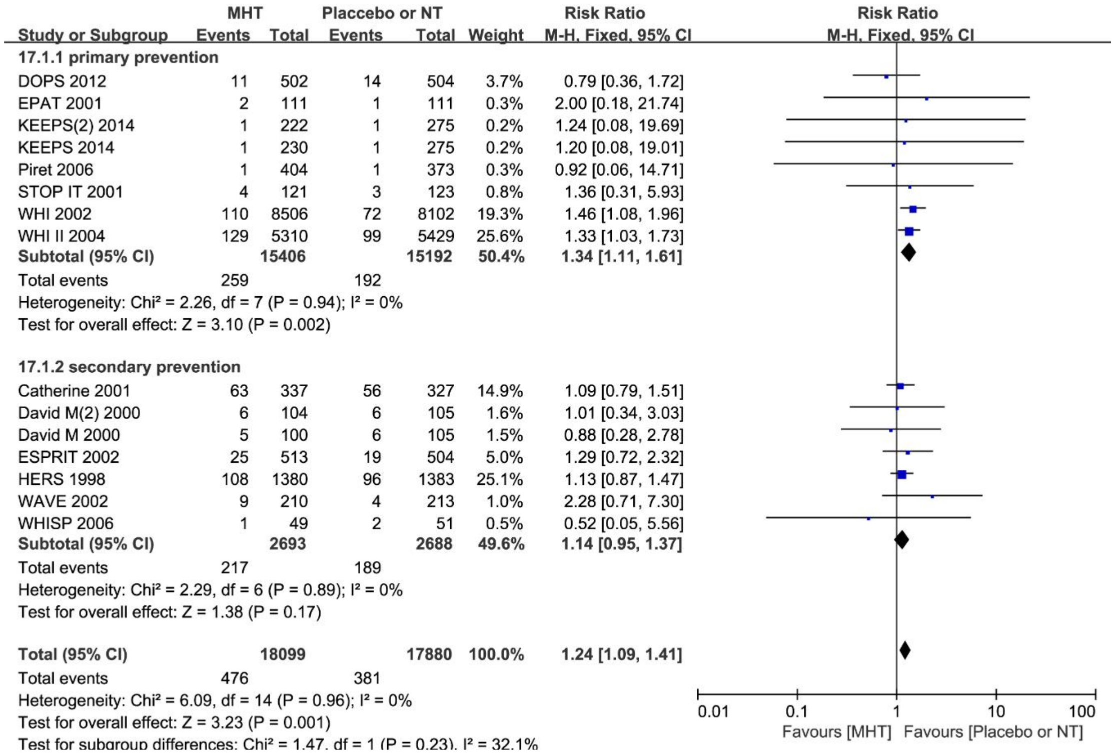

(D)

Study or Subgroup	MHT Events	MHT Total	Placebo(or NT) Events	Placebo(or NT) Total	Weight	Risk Ratio M-H, Fixed, 95% CI
**20.1.1 primary prevention**						
Catherine 2001	3	337	4	327	2.2%	0.73 [0.16, 3.23]
DOPS 2012	2	502	2	504	1.1%	1.00 [0.14, 7.10]
Greenspan 2005	2	187	1	186	0.5%	1.99 [0.18, 21.75]
KEEPS(2) 2014	2	222	2	275	1.0%	1.24 [0.18, 8.72]
KEEPS 2014	1	230	2	275	1.0%	0.60 [0.05, 6.55]
STOP IT 2001	2	121	1	123	0.5%	2.03 [0.19, 22.13]
WHI 2002	151	8506	67	8102	37.2%	2.15 [1.61, 2.86]
WHI II 2004	101	5310	78	5429	41.8%	1.32 [0.99, 1.77]
WISDOM 2007	22	2196	3	2189	1.6%	7.31 [2.19, 24.39]
**Subtotal (95% CI)**		**17611**		**17410**	**87.0%**	**1.77 [1.46, 2.14]**
Total events	286		160			
Heterogeneity: Chi² = 13.47, df = 8 (P = 0.10); I² = 41%						
Test for overall effect: Z = 5.80 (P < 0.00001)						
**20.1.2 secondary prevention**						
David M(2) 2000	2	104	1	105	0.5%	2.02 [0.19, 21.93]
David M 2000	5	100	1	105	0.5%	5.25 [0.62, 44.16]
ESPRIT 2002	5	513	4	504	2.2%	1.23 [0.33, 4.55]
EVTET 2000	8	71	1	69	0.6%	7.77 [1.00, 60.53]
HERS 1998	34	1380	12	1383	6.5%	2.84 [1.48, 5.46]
WAVE 2002	4	210	4	213	2.2%	1.01 [0.26, 4.00]
WHISP 2006	1	49	1	51	0.5%	1.04 [0.07, 16.18]
**Subtotal (95% CI)**		**2427**		**2430**	**13.0%**	**2.46 [1.54, 3.95]**
Total events	59		24			
Heterogeneity: Chi² = 4.97, df = 6 (P = 0.55); I² = 0%						
Test for overall effect: Z = 3.75 (P = 0.0002)						
**Total (95% CI)**		**20038**		**19840**	**100.0%**	**1.86 [1.56, 2.22]**
Total events	345		184			
Heterogeneity: Chi² = 19.73, df = 15 (P = 0.18); I² = 24%						
Test for overall effect: Z = 6.82 (P < 0.00001)						
Test for subgroup differences: Chi² = 1.64, df = 1 (P = 0.20), I² = 38.9%						

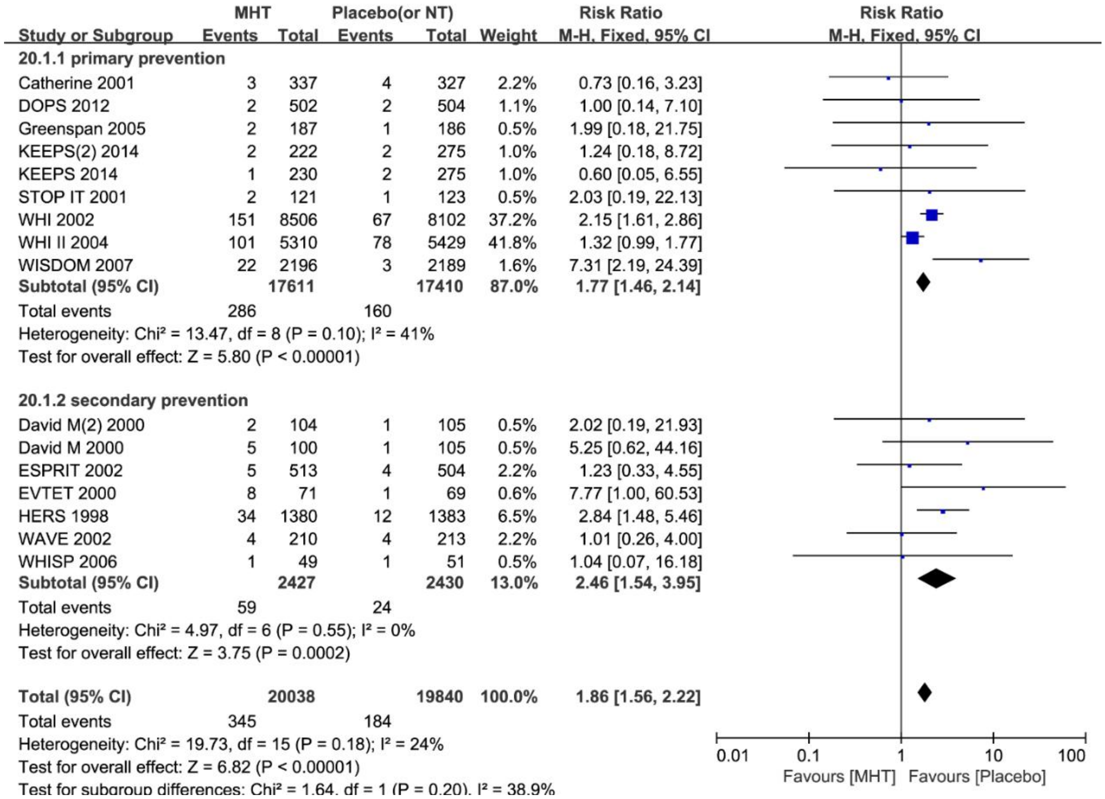

Figure S6C: Effect of different MHT prevention modes on stroke; Figure S6D: Effect of different MHT prevention modes on venous thromboembolism

(E)

Study or Subgroup	MHT Mean	MHT SD	MHT Total	Placebo(or NT) Mean	Placebo(or NT) SD	Placebo(or NT) Total	Weight	Std. Mean Difference IV, Random, 95% CI
**23.1.1 primary prevention**								
Andrew(2) 2007	4.08	4.44	17	4.51	4.46	17	6.0%	-0.09 [-0.77, 0.58]
Andrew 2007	4.54	4.55	17	4.51	4.46	17	6.0%	0.01 [-0.67, 0.68]
Claire 2007	4.7	4.8	9	6.2	4.7	9	5.5%	-0.30 [-1.23, 0.63]
Enderle 2000	5	2.8	10	3.5	1.7	10	5.6%	0.62 [-0.28, 1.52]
J.E.B 2003	3.4	0.45	9	0.5	0.03	9	1.8%	8.66 [5.33, 11.99]
Mark K 2005	4.1	6.5	28	4.1	5.2	33	6.3%	0.00 [-0.50, 0.50]
**Subtotal (95% CI)**			**90**			**95**	**31.1%**	**0.44 [-0.36, 1.23]**
Heterogeneity: Tau² = 0.72; Chi² = 27.89, df = 5 (P < 0.0001); I² = 82%								
Test for overall effect: Z = 1.07 (P = 0.28)								
**23.1.2 secondary prevention**								
Akihiko(2) 2004	7.5	3.3	15	4.9	3.6	14	5.8%	0.73 [-0.02, 1.49]
Akihiko 2004	8.9	3.7	15	4.9	3.6	14	5.8%	1.06 [0.28, 1.85]
Andrew(2) 2007	4.51	3.49	17	4.73	4.09	16	6.0%	-0.06 [-0.74, 0.63]
Andrew 2007	4.62	4.96	16	4.73	4.09	16	6.0%	-0.02 [-0.72, 0.67]
Antonino 2001	11.55	1.46	30	7.17	1.03	30	5.7%	3.42 [2.61, 4.23]
Aris 2012	7.59	2.63	40	4.91	2.49	40	6.3%	1.04 [0.57, 1.50]
B.G 2001	10.58	3.94	31	6.26	3.69	30	6.2%	1.12 [0.57, 1.66]
Kerrie 2013	6.2	0.6	15	5.4	0.6	10	5.6%	1.29 [0.40, 2.18]
Marie(2) 1998	9.6	0.8	9	4.7	0.6	8	2.4%	6.52 [3.84, 9.19]
Marie 1998	11.1	0.1	9	4.7	0.6	8	0.7%	14.59 [8.91, 20.27]
Paola 2011	12.75	2.57	20	9.09	2.25	20	5.9%	1.49 [0.78, 2.19]
PERT 2019	7.1	0.5	86	6.4	0.5	86	6.5%	1.39 [1.06, 1.73]
W.Marchien 1999	6.7	0.9	14	7.1	1.3	13	5.8%	-0.35 [-1.11, 0.41]
**Subtotal (95% CI)**			**317**			**305**	**68.9%**	**1.32 [0.73, 1.92]**
Heterogeneity: Tau² = 0.95; Chi² = 109.27, df = 12 (P < 0.00001); I² = 89%								
Test for overall effect: Z = 4.34 (P < 0.0001)								
**Total (95% CI)**			**407**			**400**	**100.0%**	**1.09 [0.57, 1.60]**
Heterogeneity: Tau² = 1.03; Chi² = 168.74, df = 18 (P < 0.00001); I² = 89%								
Test for overall effect: Z = 4.15 (P < 0.0001)								
Test for subgroup differences: Chi² = 3.05, df = 1 (P = 0.08), I² = 67.2%								

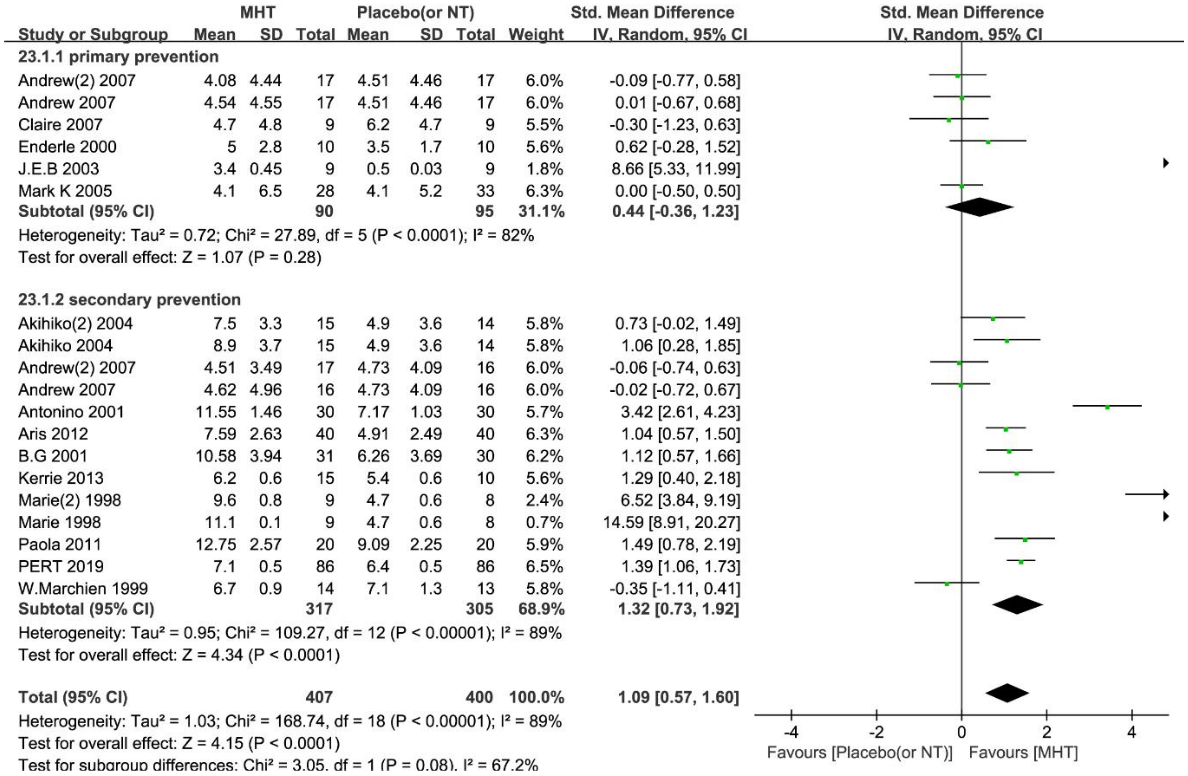

(F)

Study or Subgroup	MHT Mean	MHT SD	MHT Total	Placebo(or NT) Mean	Placebo(or NT) SD	Placebo(or NT) Total	Weight	Std. Mean Difference IV, Random, 95% CI
**21.1.1 primary prevention**								
Akihiko(2) 2004	16.9	7.7	15	16.9	3.8	14	7.5%	0.00 [-0.73, 0.73]
Akihiko 2004	18.3	3.3	15	16.9	3.8	14	7.4%	0.38 [-0.35, 1.12]
Antonino 2001	15.67	1.83	30	13.58	1.81	30	8.7%	1.13 [0.59, 1.68]
Aris 2012	15.81	4.59	40	16.54	4.69	44	9.5%	-0.16 [-0.58, 0.27]
B.G 2001	20.18	7.73	31	17.87	5.7	30	9.0%	0.33 [-0.17, 0.84]
Marie(2) 1998	11.3	1.2	9	12.7	1.3	8	5.6%	-1.07 [-2.10, -0.03]
Marie 1998	12.1	0.1	9	12.7	1.3	8	5.9%	-0.64 [-1.62, 0.34]
Paola 2011	21.3	3.13	20	15.7	3.51	20	7.5%	1.65 [0.92, 2.38]
PERT 2019	30	0.9	86	30	0.9	86	10.3%	0.00 [-0.30, 0.30]
W.Marchien 1999	14.4	1.1	14	12.4	1.8	13	6.7%	1.31 [0.47, 2.16]
**Subtotal (95% CI)**			**269**			**267**	**78.3%**	**0.33 [-0.11, 0.76]**
Heterogeneity: Tau² = 0.36; Chi² = 46.44, df = 9 (P < 0.00001); I² = 81%								
Test for overall effect: Z = 1.48 (P = 0.14)								
**21.1.2 secondary prevention**								
Claire 2007	18.7	8.7	9	15.8	7.1	9	6.2%	0.35 [-0.59, 1.28]
Enderle 2000	12.6	5.7	10	11.2	6.9	10	6.5%	0.21 [-0.67, 1.09]
Mark K 2005	11.5	6.7	28	12.9	7.2	33	9.0%	-0.20 [-0.70, 0.31]
**Subtotal (95% CI)**			**47**			**52**	**21.7%**	**-0.02 [-0.41, 0.38]**
Heterogeneity: Tau² = 0.00; Chi² = 1.34, df = 2 (P = 0.51); I² = 0%								
Test for overall effect: Z = 0.08 (P = 0.94)								
**Total (95% CI)**			**316**			**319**	**100.0%**	**0.27 [-0.08, 0.62]**
Heterogeneity: Tau² = 0.29; Chi² = 49.25, df = 12 (P < 0.00001); I² = 76%								
Test for overall effect: Z = 1.54 (P = 0.12)								
Test for subgroup differences: Chi² = 1.31, df = 1 (P = 0.25), I² = 23.8%								

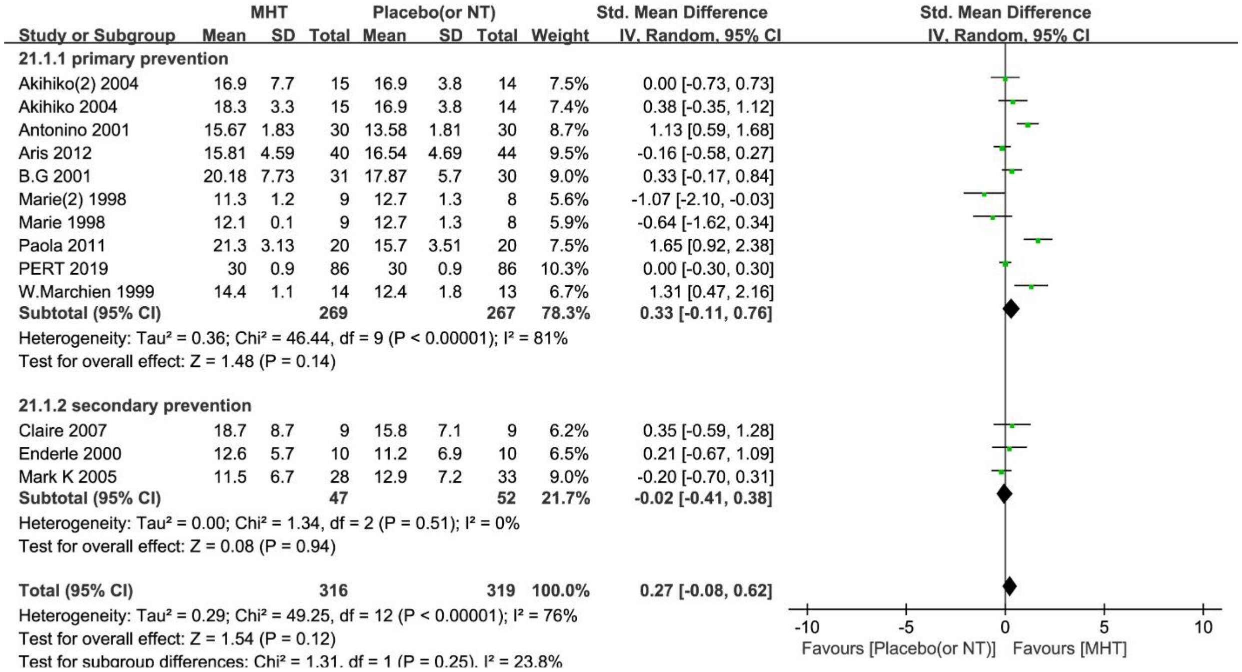

Figure S6E: Effect of different MHT prevention modes on FMD; Figure S6F: Effect of different MHT prevention modes on NMD

Figure S7 Subgroup analysis of MHT protocols

(A)

Study or Subgroup	HRT Events	HRT Total	Placebo Events	Placebo Total	Weight	Risk Ratio M-H, Fixed, 95% CI
**10.1.1 Treated by etrogen**						
Catherine 2001	48	337	41	327	5.3%	1.14 [0.77, 1.67]
David M 2000	8	100	6	105	0.8%	1.40 [0.50, 3.89]
ESPRIT 2002	32	513	61	504	7.9%	0.52 [0.34, 0.78]
KEEPS(2) 2014	1	222	1	275	0.1%	1.24 [0.08, 19.69]
KEEPS 2014	2	230	1	275	0.1%	2.39 [0.22, 26.20]
WELL-HART 2003	2	54	4	61	0.5%	0.56 [0.11, 2.96]
WHI II 2004	291	5310	289	5429	36.7%	1.03 [0.88, 1.21]
**Subtotal (95% CI)**		**6766**		**6976**	**51.4%**	**0.97 [0.84, 1.11]**
Total events	384		403			
Heterogeneity: Chi² = 11.81, df = 6 (P = 0.07); I² = 49%						
Test for overall effect: Z = 0.50 (P = 0.62)						
**10.1.2 Treated by estrogen+progesterone**						
David M(2) 2000	3	104	6	105	0.8%	0.50 [0.13, 1.97]
ERT II 1979	3	84	7	84	0.9%	0.43 [0.11, 1.60]
HERS 1998	131	1380	123	1383	15.8%	1.07 [0.84, 1.35]
Piret 2006	1	404	1	373	0.1%	0.92 [0.06, 14.71]
WAVE 2002	14	210	8	213	1.0%	1.77 [0.76, 4.14]
WELL-HART(2) 2003	3	54	4	61	0.5%	0.85 [0.20, 3.62]
WHI 2002	231	8506	218	8102	28.7%	1.01 [0.84, 1.21]
WHISP 2006	1	49	2	51	0.3%	0.52 [0.05, 5.56]
WISDOM 2007	8	2196	5	2189	0.6%	1.59 [0.52, 4.87]
**Subtotal (95% CI)**		**12987**		**12561**	**48.6%**	**1.03 [0.90, 1.18]**
Total events	395		374			
Heterogeneity: Chi² = 5.46, df = 8 (P = 0.71); I² = 0%						
Test for overall effect: Z = 0.40 (P = 0.69)						
**Total (95% CI)**		**19753**		**19537**	**100.0%**	**1.00 [0.91, 1.10]**
Total events	779		777			
Heterogeneity: Chi² = 17.65, df = 15 (P = 0.28); I² = 15%						
Test for overall effect: Z = 0.07 (P = 0.95)						
Test for subgroup differences: Chi² = 0.41, df = 1 (P = 0.52), I² = 0%						

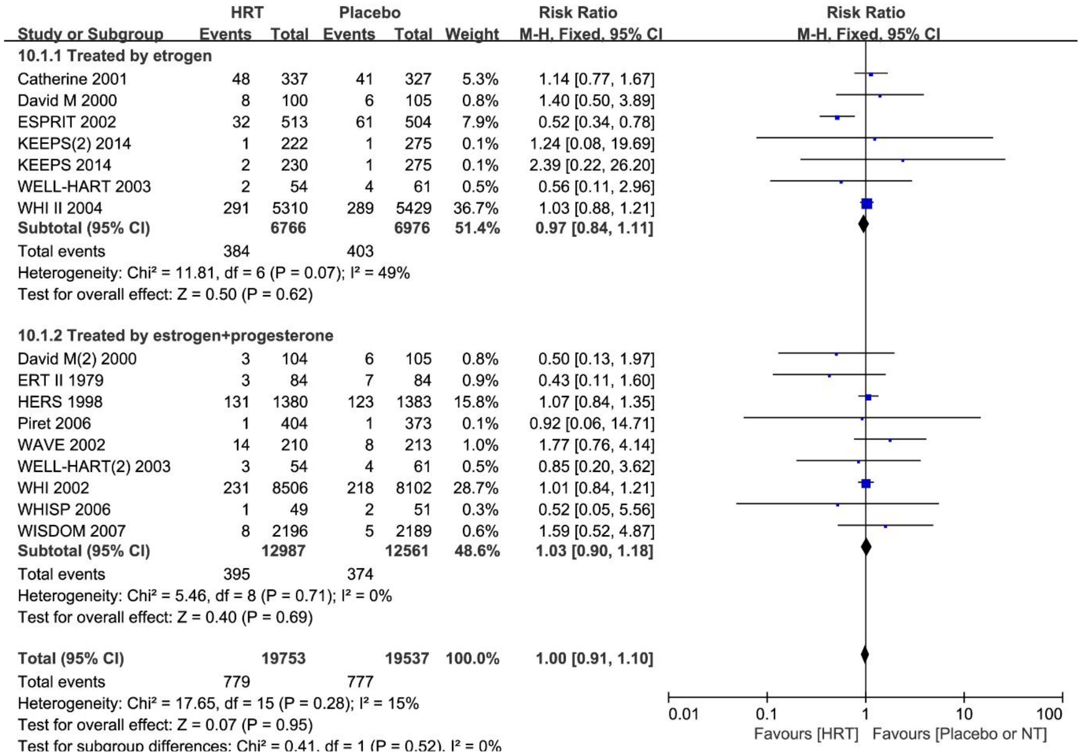

(B)

Study or Subgroup	MHT Events	MHT Total	Placebo(or NT) Events	Placebo(or NT) Total	Weight	Risk Ratio M-H, Fixed, 95% CI
**13.2.1 Treated by estrogen**						
Catherine 2001	25	337	25	327	3.6%	0.97 [0.57, 1.65]
David M 2000	10	100	10	105	1.4%	1.05 [0.46, 2.41]
ESPRIT 2002	62	513	61	504	8.6%	1.00 [0.72, 1.39]
WHI II 2004	177	5310	199	5429	27.5%	0.91 [0.75, 1.11]
**Subtotal (95% CI)**		**6260**		**6365**	**41.1%**	**0.94 [0.80, 1.10]**
Total events	274		295			
Heterogeneity: Chi² = 0.32, df = 3 (P = 0.96); I² = 0%						
Test for overall effect: Z = 0.79 (P = 0.43)						
**13.2.2 Treated by estrogen+progesterone**						
David M(2) 2000	8	104	10	105	1.4%	0.81 [0.33, 1.97]
ERT II 1979	1	84	3	84	0.4%	0.33 [0.04, 3.14]
HERS 1998	192	1380	193	1383	27.0%	1.00 [0.83, 1.20]
WAVE 2002	12	210	10	213	1.4%	1.22 [0.54, 2.76]
WHI II 2004	177	5310	199	5429	27.5%	0.91 [0.75, 1.11]
WHISP 2006	3	49	8	51	1.1%	0.39 [0.11, 1.39]
WISDOM 2007	5	2196	1	2189	0.1%	4.98 [0.58, 42.63]
**Subtotal (95% CI)**		**9333**		**9454**	**58.9%**	**0.95 [0.83, 1.08]**
Total events	398		424			
Heterogeneity: Chi² = 5.95, df = 6 (P = 0.43); I² = 0%						
Test for overall effect: Z = 0.76 (P = 0.44)						
**Total (95% CI)**		**15593**		**15819**	**100.0%**	**0.95 [0.85, 1.05]**
Total events	672		719			
Heterogeneity: Chi² = 6.28, df = 10 (P = 0.79); I² = 0%						
Test for overall effect: Z = 1.09 (P = 0.28)						
Test for subgroup differences: Chi² = 0.02, df = 1 (P = 0.90), I² = 0%						

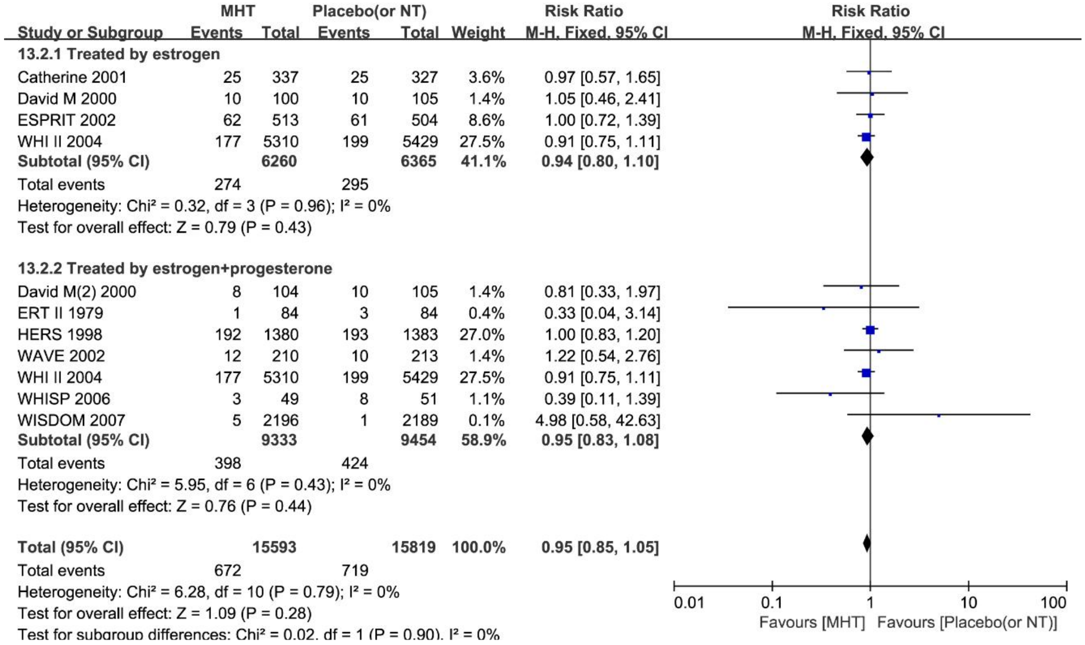

Figure S7A: Effect of different MHT protocols on all-cause death; Figure S7B: Effect of different MHT protocols on cardiovascular events

(C)

Study or Subgroup	MHT Events	MHT Total	Placebo(or NT) Events	Placebo(or NT) Total	Weight	Risk Ratio M-H, Fixed, 95% CI
**16.1.1 Treated by estrogen**						
Catherine 2001	63	337	56	327	15.6%	1.09 [0.79, 1.51]
David M 2000	5	100	6	105	1.6%	0.88 [0.28, 2.78]
EPAT 2001	2	111	1	111	0.3%	2.00 [0.18, 21.74]
ESPRIT 2002	25	513	19	504	5.2%	1.29 [0.72, 2.32]
KEEPS(2) 2014	1	222	1	275	0.2%	1.24 [0.08, 19.69]
KEEPS 2014	1	230	1	275	0.2%	1.20 [0.08, 19.01]
WHI II 2004	129	5310	99	5429	26.8%	1.33 [1.03, 1.73]
**Subtotal (95% CI)**		**6823**		**7026**	**50.0%**	**1.24 [1.03, 1.50]**
Total events	226		183			
Heterogeneity: Chi² = 1.41, df = 6 (P = 0.97); I² = 0%						
Test for overall effect: Z = 2.25 (P = 0.02)						
**16.1.2 Treated by estrogen+progesterone**						
David M(2) 2000	6	104	6	105	1.6%	1.01 [0.34, 3.03]
HERS 1998	108	1380	96	1383	26.3%	1.13 [0.87, 1.47]
Piret 2006	1	404	1	373	0.3%	0.92 [0.06, 14.71]
WAVE 2002	9	210	4	213	1.1%	2.28 [0.71, 7.30]
WHI 2002	110	8506	72	8102	20.2%	1.46 [1.08, 1.96]
WHISP 2006	1	49	2	51	0.5%	0.52 [0.05, 5.56]
**Subtotal (95% CI)**		**10653**		**10227**	**50.0%**	**1.27 [1.05, 1.54]**
Total events	235		181			
Heterogeneity: Chi² = 3.34, df = 5 (P = 0.65); I² = 0%						
Test for overall effect: Z = 2.50 (P = 0.01)						
**Total (95% CI)**		**17476**		**17253**	**100.0%**	**1.26 [1.10, 1.44]**
Total events	461		364			
Heterogeneity: Chi² = 4.78, df = 12 (P = 0.96); I² = 0%						
Test for overall effect: Z = 3.36 (P = 0.0008)						
Test for subgroup differences: Chi² = 0.04, df = 1 (P = 0.85), I² = 0%						

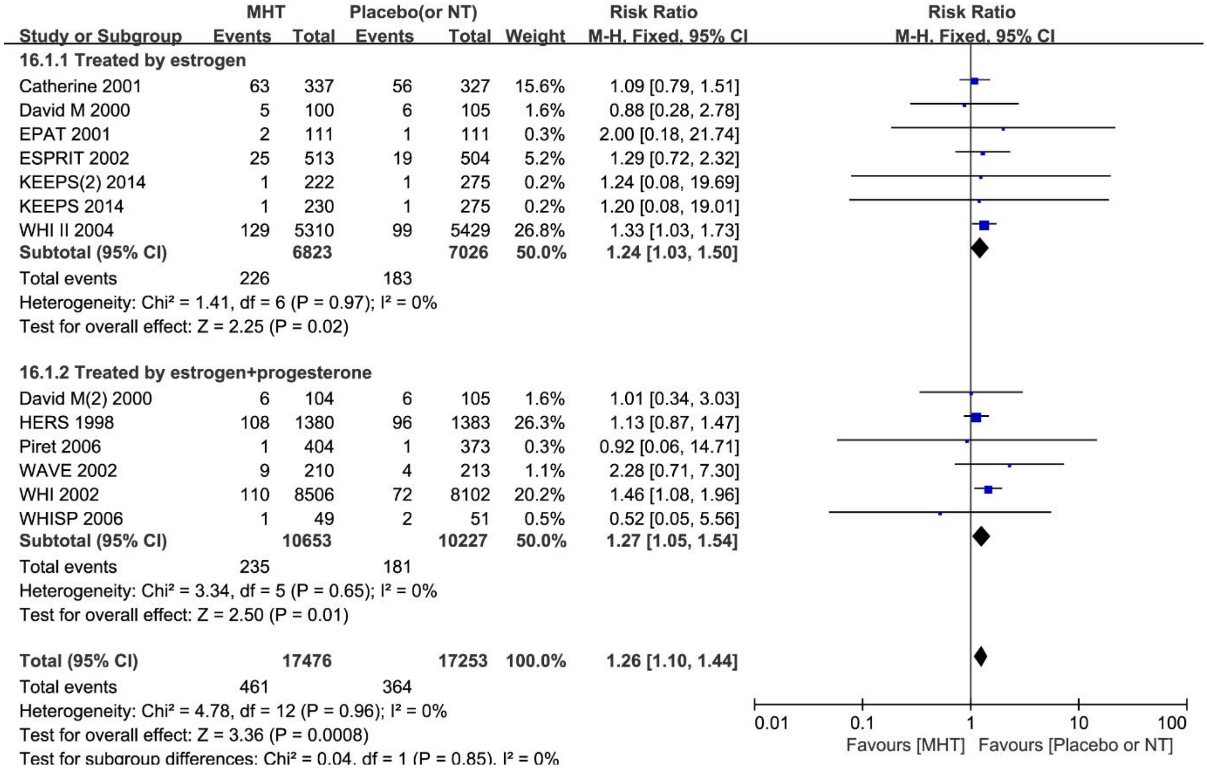

(D)

Study or Subgroup	MHT Events	MHT Total	Placebo(or NT) Events	Placebo(or NT) Total	Weight	Risk Ratio M-H, Fixed, 95% CI
**19.1.1 Treated by estrogen**						
Catherine 2001	3	337	4	327	2.4%	0.73 [0.16, 3.23]
David M 2000	5	100	1	105	0.6%	5.25 [0.62, 44.16]
ESPRIT 2002	5	513	4	504	2.3%	1.23 [0.33, 4.55]
KEEPS(2) 2014	2	222	2	275	1.0%	1.24 [0.18, 8.72]
KEEPS 2014	1	230	2	275	1.1%	0.60 [0.05, 6.55]
WHI II 2004	151	8506	67	8102	39.9%	2.15 [1.61, 2.86]
**Subtotal (95% CI)**		**9908**		**9588**	**47.3%**	**2.01 [1.54, 2.62]**
Total events	167		80			
Heterogeneity: Chi² = 4.54, df = 5 (P = 0.47); I² = 0%						
Test for overall effect: Z = 5.17 (P < 0.00001)						
**19.1.2 Treated by estrogen+progesterone**						
David M(2) 2000	2	104	1	105	0.6%	2.02 [0.19, 21.93]
EVTET 2000	8	71	1	69	0.6%	7.77 [1.00, 60.53]
HERS 1998	34	1380	12	1383	7.0%	2.84 [1.48, 5.46]
WAVE 2002	4	210	4	213	2.3%	1.01 [0.26, 4.00]
WHI 2002	151	8506	67	8102	39.9%	2.15 [1.61, 2.86]
WHISP 2006	1	49	1	51	0.6%	1.04 [0.07, 16.18]
WISDOM 2007	22	2196	3	2189	1.7%	7.31 [2.19, 24.39]
**Subtotal (95% CI)**		**12516**		**12112**	**52.7%**	**2.41 [1.89, 3.08]**
Total events	222		89			
Heterogeneity: Chi² = 7.29, df = 6 (P = 0.30); I² = 18%						
Test for overall effect: Z = 7.05 (P < 0.00001)						
**Total (95% CI)**		**22424**		**21700**	**100.0%**	**2.22 [1.86, 2.66]**
Total events	389		169			
Heterogeneity: Chi² = 12.46, df = 12 (P = 0.41); I² = 4%						
Test for overall effect: Z = 8.71 (P < 0.00001)						
Test for subgroup differences: Chi² = 0.96, df = 1 (P = 0.33), I² = 0%						

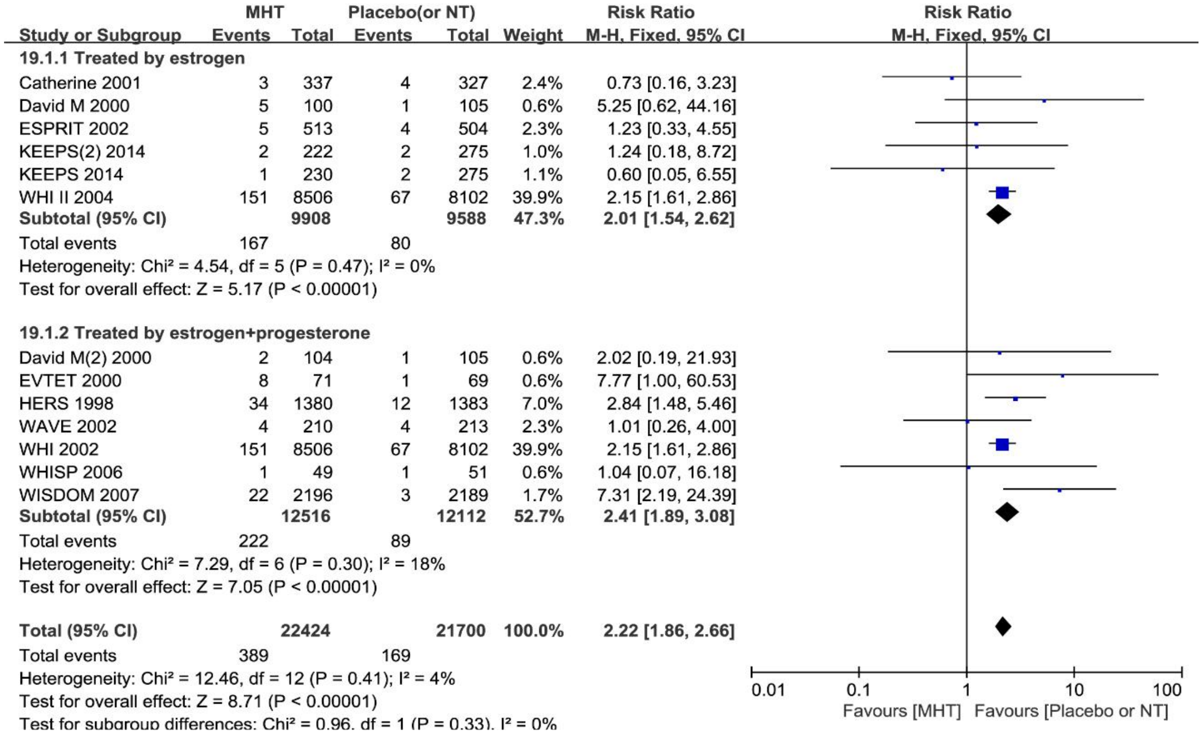

Figure S7C: Effect of different MHT protocols on stroke; Figure S7D: Effect of different MHT protocols on venous thromboembolism

(E)

Study or Subgroup	MHT Mean	MHT SD	MHT Total	Placebo(or NT) Mean	Placebo(or NT) SD	Placebo(or NT) Total	Weight	Std. Mean Difference IV, Random, 95% CI
**24.1.1 Treated by estrogen**								
Akihiko(2) 2004	7.5	3.3	15	4.9	3.6	14	5.8%	0.73 [-0.02, 1.49]
Akihiko 2004	8.9	3.7	15	4.9	3.6	14	5.8%	1.06 [0.28, 1.85]
Andrew(2) 2007	4.54	4.55	17	4.51	4.46	17	6.0%	0.01 [-0.67, 0.68]
Andrew 2007	4.62	4.96	16	4.73	4.09	16	6.0%	-0.02 [-0.72, 0.67]
Enderle 2000	5	2.8	10	3.5	1.7	10	5.6%	0.62 [-0.28, 1.52]
J.E.B 2003	3.4	0.45	9	0.5	0.03	9	1.8%	8.66 [5.33, 11.99]
Kerrie 2013	6.2	0.6	15	5.4	0.6	10	5.6%	1.29 [0.40, 2.18]
Marie 1998	11.1	0.1	9	4.7	0.6	8	0.7%	14.59 [8.91, 20.27]
**Subtotal (95% CI)**			**106**			**98**	**37.2%**	**1.43 [0.44, 2.41]**
Heterogeneity: Tau² = 1.49; Chi² = 55.63, df = 7 (P < 0.00001); I² = 87%								
Test for overall effect: Z = 2.84 (P = 0.004)								
**24.1.2 Treated by estrogen+progesterone**								
Andrew(2) 2007	4.08	4.44	17	4.51	4.46	17	6.0%	-0.09 [-0.77, 0.58]
Andrew 2007	4.51	3.49	16	4.73	4.09	17	6.0%	-0.06 [-0.74, 0.63]
Antonino 2001	11.55	1.46	30	7.17	1.03	30	5.7%	3.42 [2.61, 4.23]
Aris 2012	7.59	2.63	40	4.91	2.49	40	6.3%	1.04 [0.57, 1.50]
B.G 2001	10.58	3.94	31	6.26	3.69	30	6.2%	1.12 [0.57, 1.66]
Claire 2007	4.7	4.8	9	6.2	4.7	9	5.5%	-0.30 [-1.23, 0.63]
Marie(2) 1998	9.6	0.8	9	4.7	0.6	8	2.4%	6.52 [3.84, 9.19]
Mark K 2005	4.1	6.5	28	4.1	5.2	33	6.3%	0.00 [-0.50, 0.50]
Paola 2011	12.75	2.57	20	9.09	2.25	20	5.9%	1.49 [0.78, 2.19]
PERT 2019	7.1	0.5	86	6.4	0.5	86	6.5%	1.39 [1.06, 1.73]
W.Marchien 1999	6.7	0.9	14	7.1	1.3	13	5.8%	-0.35 [-1.11, 0.41]
**Subtotal (95% CI)**			**300**			**303**	**62.8%**	**0.98 [0.35, 1.61]**
Heterogeneity: Tau² = 0.97; Chi² = 111.00, df = 10 (P < 0.00001); I² = 91%								
Test for overall effect: Z = 3.04 (P = 0.002)								
**Total (95% CI)**			**406**			**401**	**100.0%**	**1.09 [0.57, 1.60]**
Heterogeneity: Tau² = 1.03; Chi² = 168.73, df = 18 (P < 0.00001); I² = 89%								
Test for overall effect: Z = 4.15 (P < 0.0001)								
Test for subgroup differences: Chi² = 0.56, df = 1 (P = 0.46), I² = 0%								

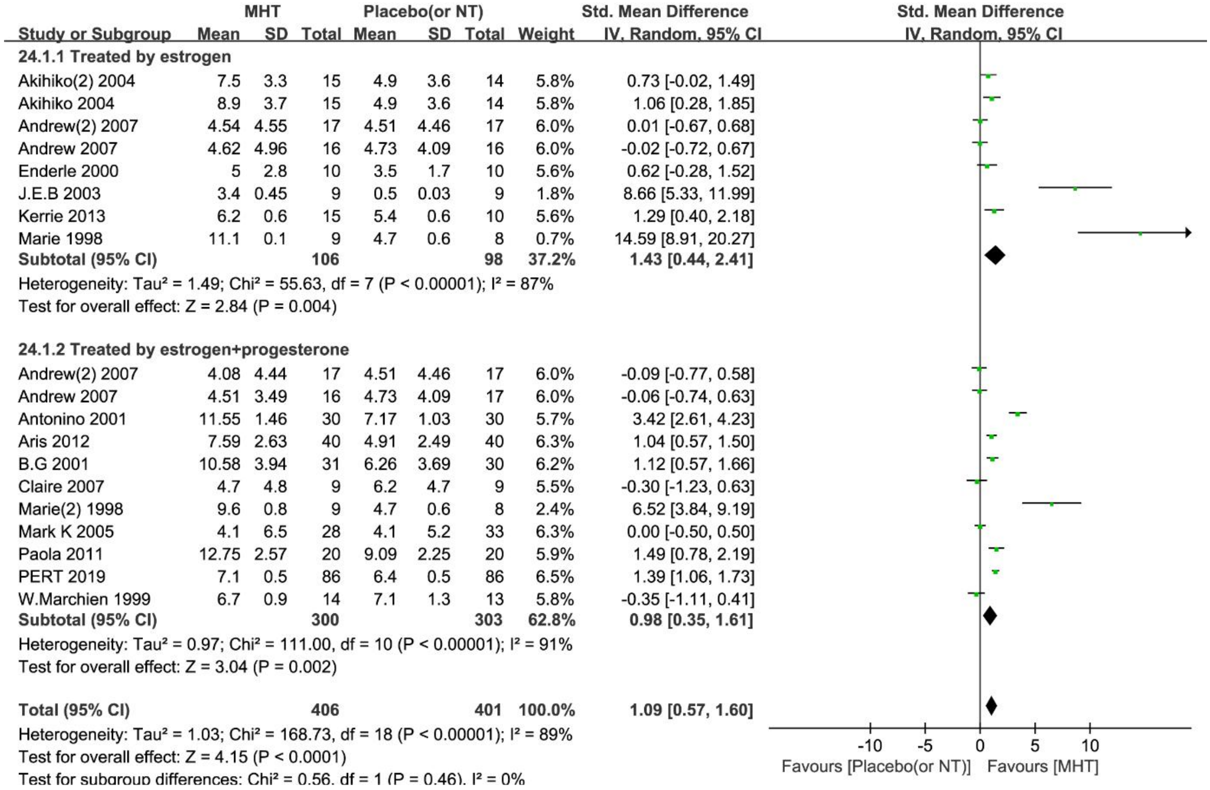

(F)

Study or Subgroup	MHT Mean	MHT SD	MHT Total	Placebo(or NT) Mean	Placebo(or NT) SD	Placebo(or NT) Total	Weight	Std. Mean Difference IV, Random, 95% CI
**26.1.1 Treated by estrogen**								
Akihiko(2) 2004	16.9	7.7	15	16.9	3.8	14	6.0%	0.00 [-0.73, 0.73]
Akihiko 2004	18.3	3.3	15	16.9	3.8	14	5.9%	0.38 [-0.35, 1.12]
Andrew(2) 2007	13.71	8.3	12	13.35	9.35	12	5.6%	0.04 [-0.76, 0.84]
Andrew 2007	20.85	9.57	6	20.25	8.91	6	3.9%	0.06 [-1.07, 1.19]
Enderle 2000	12.6	5.7	10	11.2	6.9	10	5.1%	0.21 [-0.67, 1.09]
Marie 1998	12.1	0.1	9	12.7	1.3	8	4.6%	-0.64 [-1.62, 0.34]
**Subtotal (95% CI)**			**67**			**64**	**31.1%**	**0.05 [-0.29, 0.40]**
Heterogeneity: Tau² = 0.00; Chi² = 2.82, df = 5 (P = 0.73); I² = 0%								
Test for overall effect: Z = 0.29 (P = 0.77)								
**26.1.2 Treated by estrogen+progesterone**								
Andrew(2) 2007	14.69	7.2	12	13.35	9.35	12	5.5%	0.16 [-0.65, 0.96]
Andrew 2007	21.84	6.54	7	20.25	8.91	6	4.1%	0.19 [-0.90, 1.29]
Antonino 2001	15.67	1.83	30	13.58	1.81	30	7.2%	1.13 [0.59, 1.68]
Aris 2012	15.81	4.59	40	16.54	4.69	44	8.0%	-0.16 [-0.58, 0.27]
B.G 2001	20.18	7.73	31	17.87	5.7	30	7.5%	0.33 [-0.17, 0.84]
Claire 2007	18.7	8.7	9	15.8	7.1	9	4.8%	0.35 [-0.59, 1.28]
Marie(2) 1998	11.3	1.2	9	12.7	1.3	8	4.3%	-1.07 [-2.10, -0.03]
Mark K 2005	11.5	6.7	28	12.9	7.2	33	7.5%	-0.20 [-0.70, 0.31]
Paola 2011	21.3	3.13	20	15.7	3.51	20	6.0%	1.65 [0.92, 2.38]
PERT 2019	30	0.9	86	30	0.9	86	8.8%	0.00 [-0.30, 0.30]
W.Marchien 1999	14.4	1.1	14	12.4	1.8	13	5.3%	1.31 [0.47, 2.16]
**Subtotal (95% CI)**			**286**			**291**	**68.9%**	**0.34 [-0.05, 0.74]**
Heterogeneity: Tau² = 0.31; Chi² = 45.81, df = 10 (P < 0.00001); I² = 78%								
Test for overall effect: Z = 1.72 (P = 0.09)								
**Total (95% CI)**			**353**			**355**	**100.0%**	**0.24 [-0.04, 0.53]**
Heterogeneity: Tau² = 0.22; Chi² = 49.48, df = 16 (P < 0.0001); I² = 68%								
Test for overall effect: Z = 1.66 (P = 0.10)								
Test for subgroup differences: Chi² = 1.21, df = 1 (P = 0.27), I² = 17.0%								

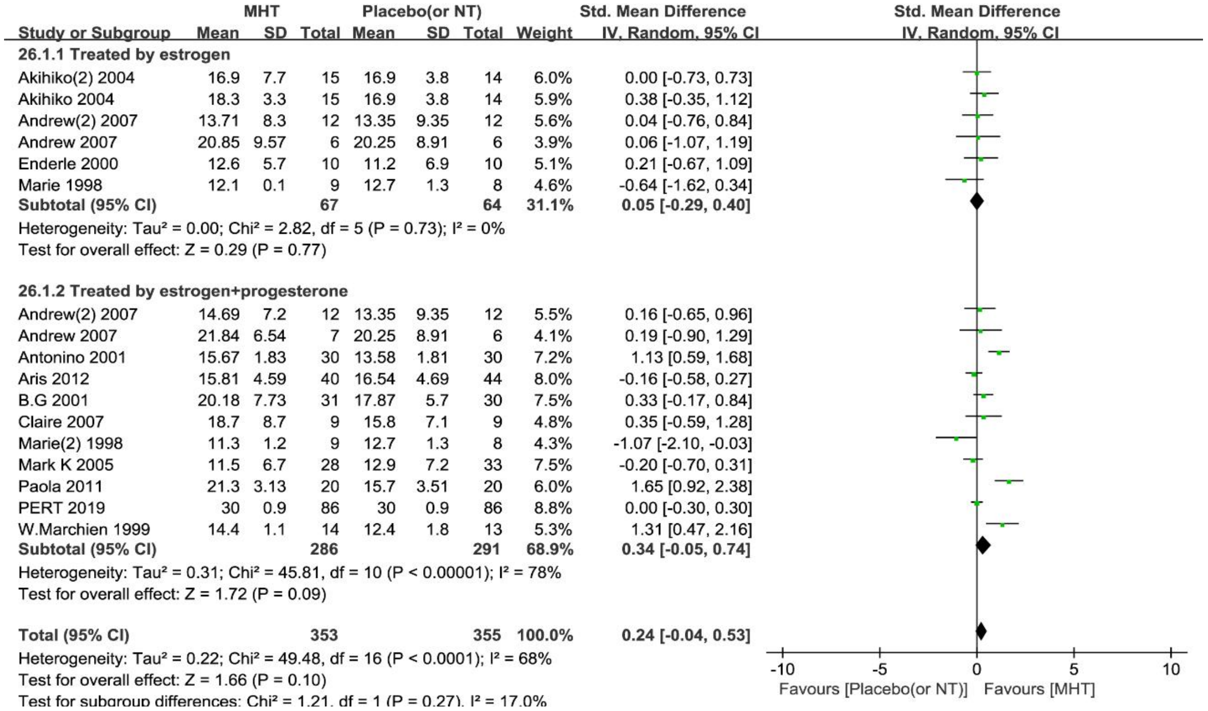

Figure S7E: Effect of different MHT protocols on FMD; Figure S7F: Effect of different MHT protocols on NMD
